# The crystal structures of the ligand *N*-(quinolin-8-yl)pyrazine-2-carboxamide and of a tetra­nuclear copper(II) complex

**DOI:** 10.1107/S2056989019005450

**Published:** 2019-05-10

**Authors:** Dilovan S. Cati, Helen Stoeckli-Evans

**Affiliations:** a Debiopharm International S.A., Chemin Messidor 5-7, CP 5911, CH-1002 Lausanne, Switzerland; b Institute of Physics, University of Neuchâtel, rue Emile-Argand 11, CH-2000 Neuchâtel, Switzerland

**Keywords:** crystal structure, pyrazine, quinoline, carboxamide, copper(II), tetra­nuclear complex, paddle-wheel, hydrogen bonding, offset π–π inter­actions, Hirshfeld surface analysis, supra­molecular framework

## Abstract

The title tridentate ligand, C_14_H_10_N_4_O, *N*-(quinolin-8-yl)pyrazine-2-carboxamide (**HL1**), crystallizes with three independent mol­ecules (*A*, *B* and *C*) in the asymmetric unit. All three mol­ecules are relatively planar (r.m.s. deviations are 0.068, 0.055 and 0.06 Å, respectively), with the NH H atom forming three-centered (bifurcated) intra­molecular N—H⋯N hydrogen bonds in each mol­ecule. There is also an intra­molecular C—H⋯O contact present in each mol­ecule, involving the benzene ring of the quinoline unit and the amide carboxamide O atom. In the crystal, the three mol­ecules stack in columns with the various mol­ecules being linked by offset π-π inter­actions [inter­centroid distances vary from 3.367 (5) to 3.589 (5) Å], forming layers parallel to the *ab* plane. The title complex, [Cu_4_(C_42_H_44_N_8_O_16_)]·2CH_3_OH, {hexa-μ-acetato-1:2κ^2^ *O*:*O*′;2:3κ^8^ *O*:*O*′;3:4κ^2^ *O*:*O*′-di­methanol-1κ*O*,2κ*O*-bis­[*N*-(quinolin-8-yl)pyrazine-2-carboxamide]-1κ^3^ *N*,*N*′,*N*′′;4κ^3^ *N*,*N*′,*N*′′-tetra­copper(II) methanol disolvate} (**I**), was obtained by the reaction of **HL1** with Cu(CH_3_CO_2_)_2_. It consists of a tetra­nuclear complex with a central tetra­kis­(μ-acetato)­dicopper paddle-wheel moiety linked on either side *via* bridging acetato ions to a mononuclear copper(II)–(**L1**) complex; it crystallizes as a methanol disolvate. The complex possesses inversion symmetry, being located about a center of symmetry situated at the mid-point of the Cu⋯Cu bond of the paddle-wheel moiety. In the crystal, the complex mol­ecules are linked by O—H⋯O hydrogen bonds, forming chains along the [01] direction, which are linked by offset π–π inter­actions [inter­centroid distance = 3.7367 (11) Å] and C—H⋯O hydrogen bonds, leading to the formation of a supra­molecular framework.

## Abstract 2

The title tridentate ligand (**HL1**), crystallizes with three independent mol­ecules in the asymmetric unit. Its reaction with Cu(Ac)_2_ produced a tetra­nuclear complex with a central tetra­kis­(μ-acetato)­dicopper paddle-wheel moiety linked on either side *via* bridging acetate anions to a mononuclear copper(II)–(**L1**) complex.

## Chemical context   

The crystal structures of a number of hetero bimetallic iron–manganese cyano complexes of the ligand **HL1** have been synthesized in order to explore their super-exchange magnetic properties (Kim *et al.*, 2007 ▸; Zhou *et al.*, 2014 ▸). To the best of our knowledge (Cambridge Structural Database; Groom *et al.*, 2016 ▸), the crystal structure of the ligand itself has never been described, although the structure of the pyridine analogue, *N*-(8-quinol­yl)pyridine-2-carboxamide, has been reported (Zhang *et al.*, 2001 ▸). There is only one previous report of a copper(II) complex of ligand **HL1**, *viz*. (acetato)[*N*-(quinolin-8-yl)pyrazine-2-carboxamidato]copper(II) monohydrate, a mononuclear complex with the ligand coordinating in a tridentate fashion (Meghdadi *et al.*, 2013 ▸). It has been shown previously that pyrazine carboxamide ligands are useful for the synthesis of transition-metal complexes that exhibit magnetic super-exchange and anion encapsulation (Hausmann *et al.*, 2003 ▸; Cati *et al.*, 2004 ▸; Klingele *et al.*, 2007 ▸). During further work in this area (Cati, 2002 ▸), the title copper(II) complex, **I**, of ligand **HL1** was synthesized, and we report herein on the crystal structures of ligand **HL1** and complex **I**. The various inter­molecular inter­actions in the crystal of **HL1** have been studied by Hirshfeld surface analysis.
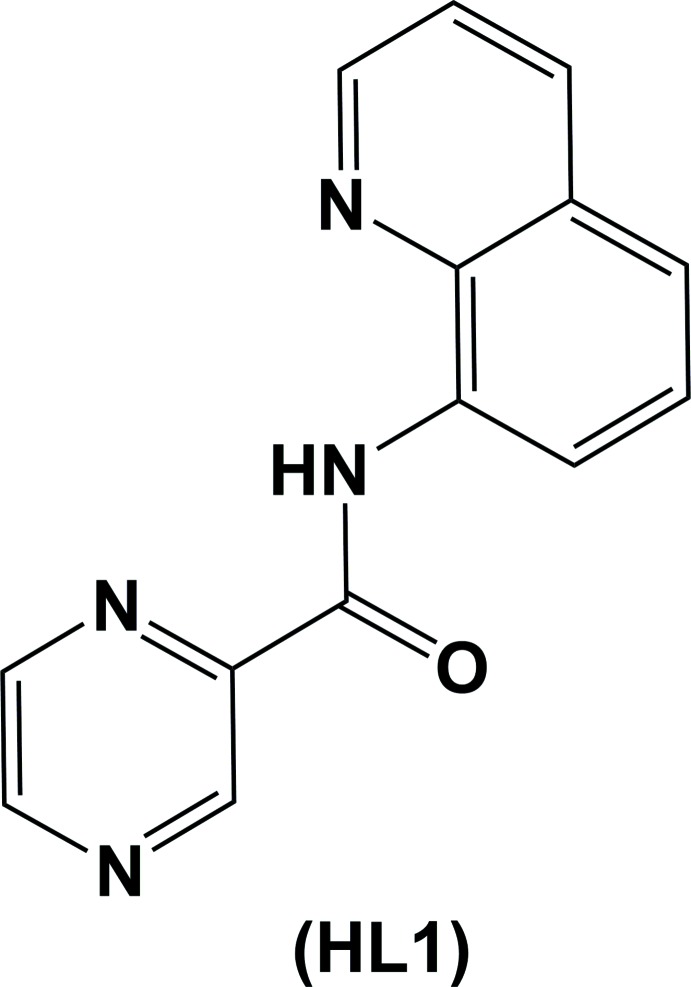
 
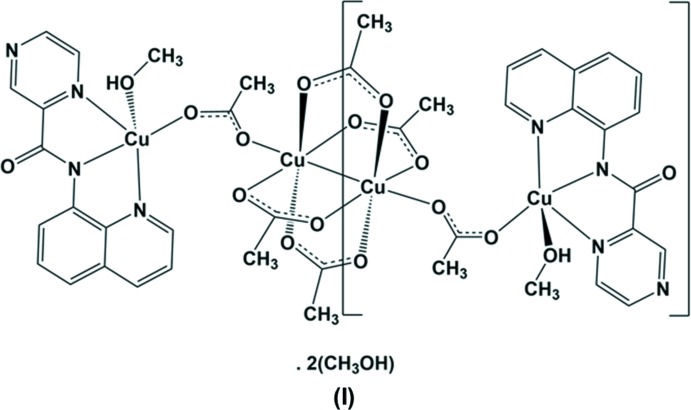


## Structural commentary   

**Figure 1:**
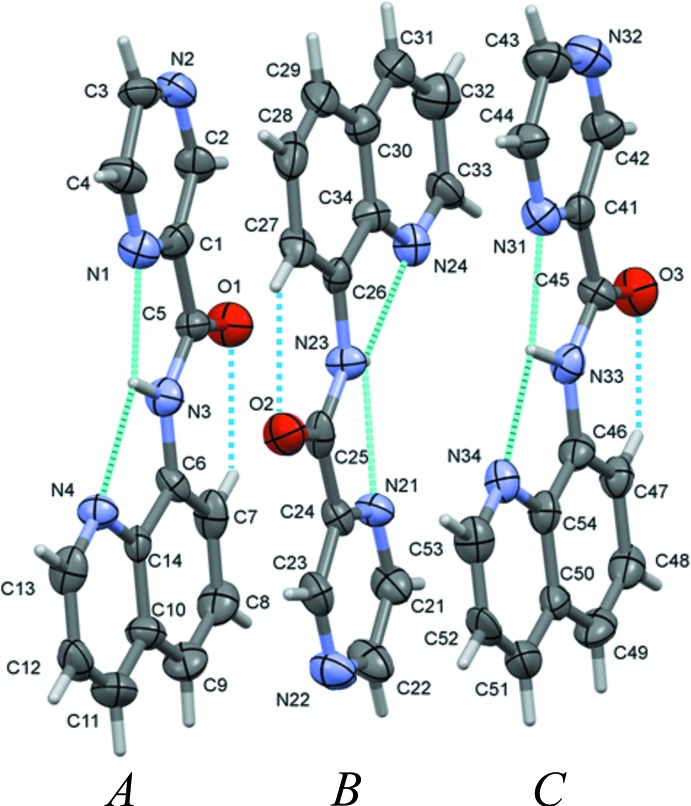
A view of the mol­ecular structure of the three independent mol­ecules (*A*, *B* and *C*) of ligand **HL1**, with the atom labelling. Displacement ellipsoids are drawn at the 50% probability level. Intra­molecular N—H⋯O and C—H⋯O contacts (see Table 1 ▸) are shown as dashed lines.

The ligand **HL1** crystallized with three independent mol­ecules (*A*, *B* and *C*) in the asymmetric unit, and their mol­ecular structures are illustrated in Fig. 1 ▸. In each mol­ecule the carboxamide NH H atom forms three-centered (bifurcated) intra­molecular N—H⋯N hydrogen bonds involving the quinoline and the adjacent pyrazine N atoms (Fig. 1 ▸ and Table 1 ▸). This arrangement is similar to that observed in 1,3-bis­(2-pyridyl­imino)­isoindoline (Schilf, 2004 ▸) and its pyrazine analogue, bis­(pyridin-2-yl)-6,7-di­hydro-pyrrolo­[3,4-*b*]pyrazine-5,7-di­imine (Posel & Stoeckli-Evans, 2018 ▸). There is also a short C—H⋯O contact present in each mol­ecule (Fig. 1 ▸ and Table 1 ▸). Hence, the three mol­ecules have similar conformations, with the pyrazine ring being inclined to the quinoline ring by 4.5 (4)° in mol­ecule *A*, 3.1 (4)° in *B* and 4.1 (4)° in *C*. For the three mol­ecules, the r.m.s. deviations for the mean planes of the non-H atoms are 0.068, 0.055 and 0.06 Å, respectively. Inverted mol­ecule *A* on mol­ecule *B* has an r.m.s. deviation of 0.054 Å for the 19 non-H atoms, while inverted mol­ecule *B* on mol­ecule *C* has an r.m.s. deviation of 0.054 Å, and mol­ecule *A* and mol­ecule *C* have an r.m.s. deviation of 0.057 Å.

**Figure 2:**
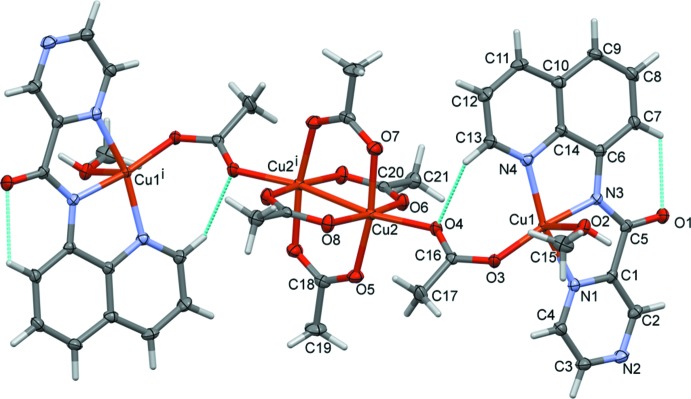
A view of the mol­ecular structure of complex **I**, with the atom labelling. Displacement ellipsoids are drawn at the 50% probability level. The unlabelled atoms are related to labelled atoms by inversion symmetry [symmetry code: (i) −*x* + 1, −*y*, −*z* + 1]. The intramolecular C-H...O contacts are shown as dashed lines (Table 3 ▸). For clarity, the methanol solvate molecules have been omitted.

Reaction of **HL1** with Cu(CH_3_CO_2_)_2_ produced a tetra­nuclear complex, **I**, with a central tetra­kis­(μ-acetato)-dicopper paddle-wheel moiety linked on either side *via* a bridging acetate anion to a mononuclear copper(II)–(**L1**) complex, illustrated in Fig. 2 ▸. Selected geometrical parameters are given in Table 2 ▸. The complex possesses inversion symmetry, being located about a center of symmetry situated at the mid-point of the Cu2⋯Cu2^i^ bond [2.6202 (6) Å; symmetry code: (i) −*x* + 1, −*y*, −*z* + 1)] of the paddle-wheel moiety (Table 2 ▸). Both copper atoms are fivefold coordinate; CuN_3_O_2_ for Cu1 and CuO_5_ for Cu2. Atom Cu1 is ligated in the equatorial plane by the three N atoms of the ligand and an O atom, O3, of the bridging acetate ion, and with a coordinated methanol O atom, O2, in the apical position. It has an irregular coordination sphere with a τ_5_ factor of 0.17 (τ_5_ = 0 for an ideal square-pyramidal coordination sphere, and = 1 for an ideal trigonal–pyramidal coordination sphere; Addison *et al.*, 1984 ▸). Atom Cu2 is ligated by four acetate O atoms (O5, O6, O7 and O8) of the paddle-wheel moiety in the equatorial plane and by atom O4 of the bridging acetate ion in the apical position. It has a perfect square-pyramidal coordination sphere with a τ_5_ factor of 0.01. There are two intra­molecular C—H⋯O contacts present involving the quinoline unit and oxygen atoms O1 of the carb­oxy­mide group and O4 of the bridging acetate ion (Fig. 2 ▸ and Table 3 ▸).

## Supra­molecular features   

**Figure 3:**
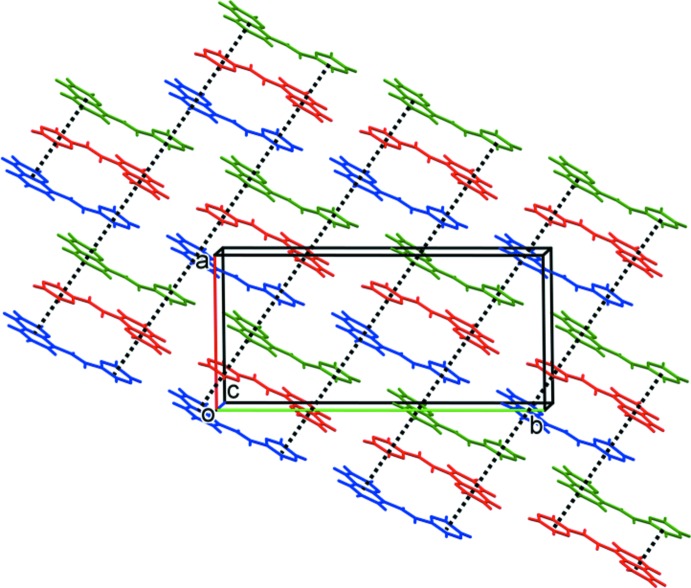
A view along the *c* axis of the crystal packing of ligand **HL1**. The π–π inter­actions, represented here by dashed lines, are given in Table 4 ▸. Colour code: *A* mol­ecules are blue, *B* are red and *C* are green.

**Figure 4:**
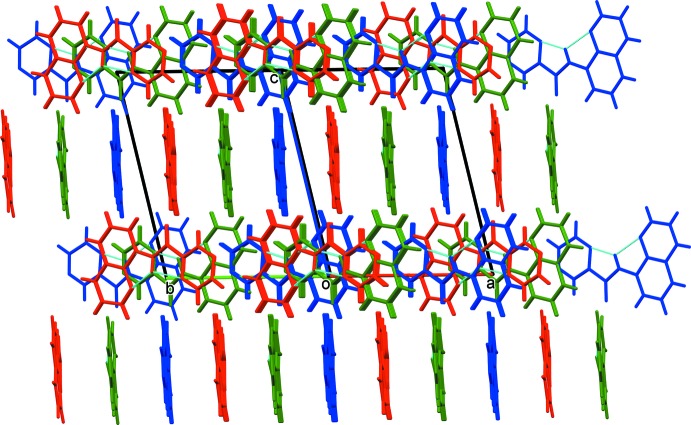
A view normal to plane (110) of the crystal packing of ligand **HL1** (colour code: *A* mol­ecules are blue, *B* are red and *C* are green).

In the crystal of ligand **HL1**, and as can be seen from Fig. 1 ▸, mol­ecule *B* is closely related to mol­ecules *A* and *C* by non-crystallographic inversion symmetry, while mol­ecules *A* and *C* are closely related by non-space group translation. An analysis with *PLATON*/ADDSYM (Spek, 2009 ▸), however, concluded that no obvious extra crystallographic symmetry was present and no change in the space group (*Cc*) was required. In the crystal, packets of the three mol­ecules stack in the order (*ABC*), (*ABC*) *etc* (Fig. 3 ▸; *A* blue, *B* red, *C* green). They are linked by offset π–π inter­actions, so forming layers lying parallel to the *ab* plane (Fig. 4 ▸ and Table 4 ▸).

**Figure 5:**
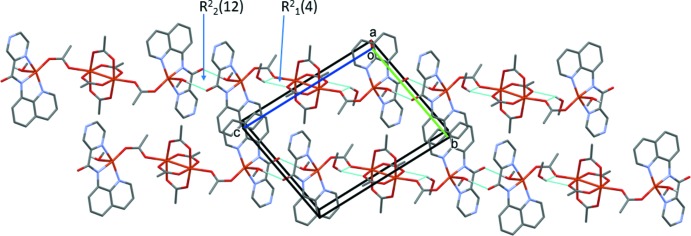
A partial view along the *a* axis of the crystal packing of complex **I**. The hydrogen bonds (see Table 3 ▸) are shown as dashed lines and C-bound H atoms have been omitted.

**Figure 6:**
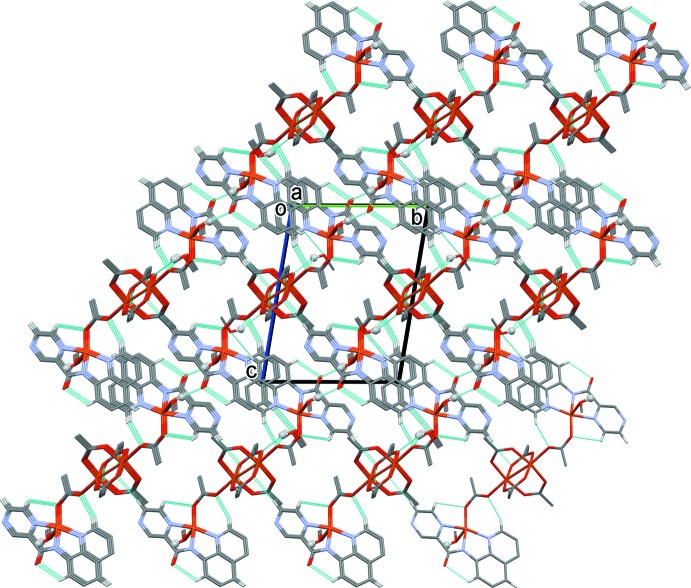
A view along the *a* axis of the crystal pack of complex **I**. The hydrogen bonds (see Table 3 ▸) are shown as dashed lines and only the H atoms involved in these inter­molecular inter­actions have been included (the two methanol hydroxyl H atoms are shown as grey balls).

In the crystal of **I**, mol­ecules are linked by pairs of O—H⋯O hydrogen bonds involving the coordinated methanol mol­ecule and the carboxamide O atom, O1, forming chains propagating along [01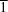]; see Table 3 ▸ and Fig. 5 ▸. The chains thus formed enclose 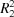
(12) ring motifs, as illustrated in Fig. 5 ▸. The methanol solvent mol­ecule is linked to the chain *via* bifurcated O—H⋯O/O hydrogen bonds, which enclose an 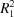
(4) ring motif (Fig. 5 ▸ and Table 3 ▸). Inversion-related chains are linked by offset π–π inter­actions involving the quinoline ring systems [Figs. 5 ▸ and 6 ▸; *Cg*⋯*Cg* ^vi^ = 3.7367 (11) Å, *Cg* is the centroid of the N4/C6–C14 ring, α = 0.04 (7)°, β = 25.7°, inter­planar distance = 3.3684 (8) Å, offset 1.618 Å; symmetry code: (vi) −*x* + 1, −*y*, −*z*]. The chains are also linked by C—H⋯O hydrogen bonds, resulting in the formation of a supra­molecular framework (Table 3 ▸ and Fig. 6 ▸).

## Hirshfeld surface analysis of ligand HL1   

The Hirshfeld surface analysis (Spackman & Jayatilaka, 2009 ▸) and the associated two-dimensional fingerprint plots (McKinnon *et al.*, 2007 ▸) were performed with *CrystalExplorer17* (Turner *et al.*, 2017 ▸). A recent article by Tiekink and collaborators (Tan *et al.*, 2019 ▸) ‘outlines the various procedures and what can be learned by using *CrystalExplorer*’.

**Figure 7:**
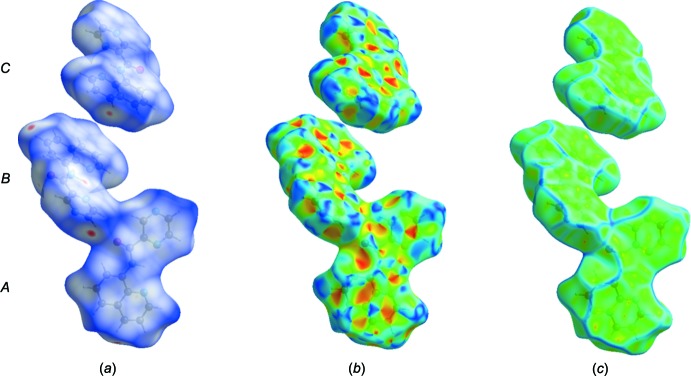
Hirshfeld surfaces for **HL1** (mol­ecules *A*, *B* and *C*) mapped over (*a*) *d*_norm_, −0.164 to 1.208 arbitrary units, (*b*) shape-index and (*c*) curvedness.

The Hirshfeld surface of **HL1** mapped over *d*_norm_ is given in Fig. 7 ▸ *a*, where short inter­atomic contacts are indicated by the faint red spots. The π–π stacking is confirmed by the small blue regions surrounding bright red spots in the various aromatic rings in Fig. 7 ▸ *b*, the Hirshfeld surface mapped over the shape-index, and by the flat regions around the aromatic regions in Fig. 7 ▸ *c*, the Hirshfeld surface mapped over the curvedness.

**Figure 8:**
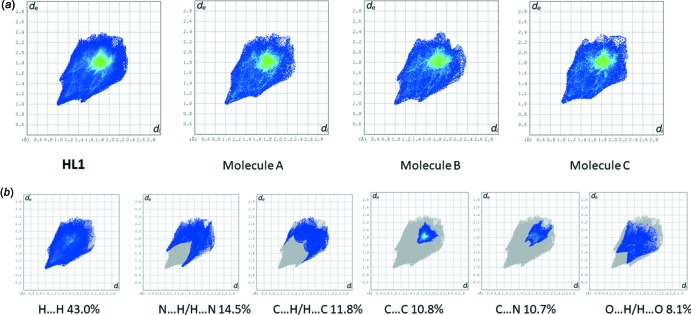
(*a*) The overall fingerprint plot for **HL1** (all three mol­ecules), and for the individual mol­ecules (*A*, *B* and *C*). (*b*) Fingerprint plots for **HL1** (all three mol­ecules) delineated into H⋯H, N⋯H/H⋯N, C⋯H/H⋯C, C⋯C, C⋯N, and O⋯H/H⋯O contacts.

The full two-dimensional fingerprint plots for **HL1** and for the individual mol­ecules are given in Fig. 8 ▸ *a*. The principal inter­molecular inter­actions for **HL1** (Fig. 8 ▸ *b*), are delineated into H⋯H at 43.0%, N⋯H/H⋯N at 14.5%, followed by C⋯H/H⋯C inter­actions at 11.8%. The contributions of the C⋯C and C⋯N inter­actions, which are 10.8 and 10.7%, respectively, are superior to the contribution of the O⋯H/H⋯O inter­actions at 8.1%. The relative percentage contributions of close contacts to the Hirshfeld surface for **HL1** and for the individual mol­ecules are similar, as indicated in Table 5 ▸.

## Database survey   

A search of the Cambridge Structural Database (Version 5.40, update February 2019; Groom *et al.*, 2016 ▸) of ligand **HL1** yielded nine hits. The majority of these compounds are hetero bimetallic iron–manganese cyano complexes that exhibit super-exchange magnetic properties [*e.g.* CSD refcodes JIVGIF and JIVGOL (Kim *et al.*, 2007 ▸) and BOLJOD, BOLJUJ and BOLKIY (Zhou *et al.*, 2014 ▸)]. Only one hit concerns a copper(II) complex, namely (acetato)(*N*-(quinolin-8-yl)pyrazine-2-carboxamidato)copper(II) monohydrate, with the ligand coordinating in a tridentate fashion (AYIFOF; Meghdadi *et al.*, 2013 ▸). The copper ion is ligated by the three N atoms of the ligand, and the two O atoms of the acetate anion, hence the copper atom is CuN_3_O_2_ five-coordinate with an irregular coordination sphere; τ_5_ = 0.17. This value is similar to that for atom Cu1 in the title complex **I** (τ_5_ factor of 0.17).

A search for complexes of the pyridine analogue of **HL1** yielded 16 hits, including the analogue itself, *N*-(8-quinol­yl)pyridine-2-carboxamide (WOVYAH; Zhang *et al.*, 2001 ▸). A number of hits involve again hetero bimetallic (Fe–Mn) cyano complexes (*e.g.* BARTUL and BARVUN; Senapati *et al.*, 2012 ▸), and trimetallic (Fe–Mn–Fe) cyano complexes (CEBYIS, CEBYOY and CEBYUE; Ni *et al.*, 2005 ▸); they all exhibit super-exchange magnetic properties. The structure of a copper(II) acetate complex, (acetato-*O*)-aqua-[*N*-(8-quinol­yl)pyridine-2-carboxamide-*N,N′,N′′*]copper(II) has also been reported (XAFKUL; Zhang *et al.*, 2007 ▸). In this mononuclear copper(II) complex, the copper ion is ligated by the three N atoms of the ligand, an O atom of the acetate anion and a water O atom, hence the copper atom is CuN_3_O_2_ five-coord­inate with an irregular coordination sphere; τ_5_ = 0.13. This geometry is similar to that of atom Cu1 in the title complex **I** (τ_5_ factor of 0.17), and that in compound AYIFOF mentioned above.

A search for the tetra­kis­(μ-acetato)­dicopper paddle-wheel moiety gave 356 hits. Limiting the search for a tetra­kis­(μ-acetato)-dicopper paddle-wheel moiety bridged on either side by an acetato group to a second copper atom gave 15 hits for 14 structures (see supporting information file S1). Eight of these compounds are polymeric structures, for example, the network structure *catena*-[octa­kis­(μ_2_-acetato-*O,O′*)[μ_2_-2,5-bis­(2-pyrid­yl)pyrazine-*N,N′,N′′,N′′′]*tetra­copper(II)] [YOM­TUP; Neels *et al.*, 1995 ▸]. Only six are tetra­nuclear compounds similar to compound **I**; for example, hexa­kis­(μ_2_-acetato)-bis­[1-(5-bromo­salicylaldimino)-3-(2-methyl­piperidino)­prop­ane]­tetra­copper(II) (PIBXOU; Chiari *et al.*, 1993 ▸), hexa­kis(μ_2_-acetato)­bis­(2-{[(2,2,6,6-tetra­methyl­piperidin-4-yl)imino] meth­yl}phenolato)tetra­copper(II) [UJOWEX; Huang & Liu, 2016 ▸], and tetra­kis­(μ_2_-acetato-*O,O′*)bis­(μ_2_-acetato-*O,O,O′*)tetra­kis­(tri­phenyl­phosphine-*P*)dicopper(I)dicopper(II) [CER­TOI; Koman *et al.*, 1984 ▸: CERTOI10; Valigura *et al.*, 1986 ▸]. The Cu⋯Cu distance in the paddle-wheel unit varies from *ca* 2.604 to 2.669 Å; in **I** this distance, Cu2⋯Cu2^i^, is 2.6201 (6) Å. The Cu⋯Cu distance involving the two copper atoms bridged by a single acetato group varies from *ca* 3.772 to 5.441 Å. The longer distance is observed when only one O atom bridges the two copper atoms as in compound **I**, where distance Cu2⋯Cu1 is *ca* 5.147 Å, close to the distance of *ca* 5.392 Å observed in UJOWEX. A shorter distance is observed when one O atom bridges the two copper atoms and the second O atom coordinates to the second copper atom, in a (μ^2^-acetato-*O,O,O′*) manner, as in CERTOI/CERTOI10 where this Cu⋯Cu distance is *ca* 3.772 Å.

## Synthesis and crystallization   

**Synthesis of** ***N*** **-(quinolin-8-yl)pyrazine-2-carboxamide (HL1):**

A suspension of pyrazine-2-carb­oxy­lic acid (1.49 g, 12 mmol) and 8-amino­quinoline (1.15 g, 8 mmol) in 80 ml of 1,2-di­chloro­ethane was distilled to azeotropically remove any solvated H_2_O (vapour temperature 355 K). The mixture was allowed to cool, and then 1,1′-carbonyl­diimidazole (1.95 g, 12 mmol) was added. After gas evolution had diminished, the solution was heated at reflux for 16 h. The reaction mixture was allowed to cool to RT and then added directly to a column (*R* = 1.2 cm, 30 g of SiO_2_) and eluted with CHCl_3_. On evaporation of the solvent the residue obtained was recrystallized from ethanol giving block-like colourless crystals of **HL1** (yield 75%, m.p. 461 K).

**Spectroscopic data for HL1** (for the numbering scheme see mol­ecule *A* in Fig. 1 ▸): ^1^H NMR (400 MHz, DMSO-*d*_6_): 11.95 (*s*, 1H, HN3); 9.41 (*d*, *J*_2,3_ = 1.4, 1H, H2); 9.02 (*m*, 2H, H3 & H4); 8.93 (*m*, 1H, H13); 8.89 (*dd*, 1H, *J*_7,8_ = 7.6, *J*_7,9_ = 1.2, H7); 8.48 (*dd*, 1H, *J*_11,12_ = 8.3, *J*_11,13_ = 1.6, H11); 7.78 (*dd*, 1H, *J*_9,8_ = 8.3, *J*_9,7_ = 1.2, H9); 7.69 (*m*, 2H, H12 & H8). ^13^C NMR (400 MHz, DMSO-*d*_6_): 161.7 (C5), 150.3 (C13), 149.2 (C4), 144.9 (C1), 144.6 (C2), 144.5 (C3), 139.0 (C14), 137.7 (C11), 134.2 (C6), 128.8 (C10), 128.0 (C8), 123.7 (C12), 123.4 (C7), 117.0 C(9). IR (KBr pellet, cm^−1^): 1686 (*vs*), 1559 (*s*), 1533 (*vs*), 1485 (*vs*), 1471 (*s*), 1460 (*s*), 1425 (*s*), 1403 (*s*), 1384 (*s*), 1325 (*s*), 1129 (*s*), 1058 (*s*), 1020 (*s*), 830 (*s*), 796 (*s*), 763 (*s*), 741 (*s*), 710 (*s*), 599 (*s*). Analysis for C_14_H_10_N_4_O (*M*_r_ = 250.26 g mol^−1^); calculated (%) C 67.19, H 4.03, N 22.39; found (%) C 67.00, H 4.04, N 22.37.

**Synthesis of compound I:**

Cu(Ac)_2_·H_2_O (74.9 mg; 0.375 mmol) was added to a solution of **HL1** (37.5 mg; 0.150 mmol) dissolved in 15 ml of methanol. The green solution was stirred at room temperature for 30 min. It was then left to allow slow evaporation of the solvent giving finally green block-like crystals of **I**. The crystals were filtered off and washed with diethyl ether (yield 61 mg, 67%). IR (KBr pellet, cm^−1^): 3422 (*s*), 1624 (*vs*), 1581 (*vs*), 1430 (*s*), 1396 (*vs*).

## Refinement   

Crystal data, data collection and structure refinement details are summarized in Table 6 ▸. Intensity data for ligand **HL1** were measured at 223 K on a four-circle diffractometer assuming a *C*-centered unit cell and only one equivalent of data were measured; hence *R*_int_ = 0 and the *h*,*k*,*l* reflections for which *h* + *k* = 2*n* + 1 were not measured. For compound **I**, data were measured at 173 K on a Stoe IPDS1, a one-circle image-plate diffractometer. For compound **I** a small cusp of data is missing. This is common with data measured using the IPDS1 for monoclinic and triclinic crystal systems.

For ligand **HL1** the NH H atoms could be located in a difference-Fourier map, but during refinement they were included in calculated positions and treated as riding: N—H = 0.87 Å with *U*_iso_(H) = 1.2*U*_eq_(N). The OH H atom of the coordinated methanol mol­ecule in complex **I** was located in a difference-Fourier map and freely refined. The OH H atom of the solvent methanol mol­ecule in **I** was included in a calculated position and treated as riding: O—H = 0.84 Å with *U*_iso_(H) = 1.5*U*_eq_(O). For **HL1** and complex **I** the C-bound H atoms were included in calculated positions and treated as riding: C—H = 0.95–0.99 Å with *U*_iso_(H) = 1.2*U*_eq_(C).

## Supplementary Material

Crystal structure: contains datablock(s) HL1, I, Global. DOI: 10.1107/S2056989019005450/zl2755sup1.cif

Structure factors: contains datablock(s) HL1. DOI: 10.1107/S2056989019005450/zl2755HL1sup2.hkl

Structure factors: contains datablock(s) I. DOI: 10.1107/S2056989019005450/zl2755Isup3.hkl

CSD search S1. DOI: 10.1107/S2056989019005450/zl2755sup4.pdf

CCDC references: 1911375, 1911374

Additional supporting information:  crystallographic information; 3D view; checkCIF report

## Figures and Tables

**Table 1:** Hydrogen-bond geometry (Å, °) for **HL1** [Chem scheme1]

*D*—H⋯*A*	*D*—H	H⋯*A*	*D*⋯*A*	*D*—H⋯*A*
N3—H3*N*⋯N1	0.87	2.21	2.661 (10)	112
N3—H3*N*⋯N4	0.87	2.23	2.667 (10)	111
C7—H7⋯O1	0.94	2.36	2.967 (13)	122
N23—H23*N*⋯N21	0.87	2.22	2.662 (10)	112
N23—H23*N*⋯N24	0.87	2.24	2.675 (11)	110
C27—H27⋯O2	0.94	2.34	2.912 (13)	119
N33—H33*N*⋯N31	0.87	2.21	2.667 (10)	113
N33—H33*N*⋯N34	0.87	2.26	2.674 (11)	109
C47—H47⋯O3	0.94	2.27	2.893 (12)	123

**Table 2:** Selected geometric parameters (Å, °) for **I** [Chem scheme1]

Cu1—N1	2.037 (2)	Cu2—O4	2.1255 (16)
Cu1—N3	1.9457 (18)	Cu2—O5	1.9703 (17)
Cu1—N4	1.998 (2)	Cu2—O6	1.9793 (15)
Cu1—O2	2.3541 (16)	Cu2—O7	1.9692 (18)
Cu1—O3	1.9401 (15)	Cu2—O8	1.9671 (16)
Cu2—Cu2^i^	2.6202 (6)		
			
O3—Cu1—N3	172.66 (8)	O7—Cu2—O5	169.04 (7)
N4—Cu1—N1	162.67 (8)	O8—Cu2—O6	168.74 (7)

Symmetry code: (i) 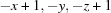.

**Table 3:** Hydrogen-bond geometry (Å, °) for **I** [Chem scheme1]

*D*—H⋯*A*	*D*—H	H⋯*A*	*D*⋯*A*	*D*—H⋯*A*
O2—H2*O*⋯O1^ii^	0.86 (3)	1.84 (3)	2.689 (3)	178 (3)
O9—H9*O*⋯O4	0.84	2.33	2.955 (3)	132
O9—H9*O*⋯O6	0.84	2.30	3.000 (3)	141
C3—H3⋯O8^iii^	0.95	2.56	3.508 (3)	174
C7—H7⋯O1	0.95	2.36	2.943 (3)	119
C9—H9⋯O9^iv^	0.95	2.57	3.415 (3)	149
C13—H13⋯O4	0.95	2.54	3.175 (3)	124
C21—H21*C*⋯O8^v^	0.98	2.59	3.563 (3)	170

Symmetry codes: (ii) 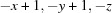; (iii) 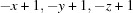; (iv) 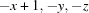; (v) 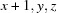.

**Table 4:** π–π inter­actions (Å, °) in the crystal of ligand **HL1** *Cg*1, *Cg*5 and *Cg*9 are the centroids of the pyrazine rings (N1/N2/C1–C4) in mol­ecule *A*, (N22/N23/C21–C24) in mol­ecule *B* and (N31/N32/C41–C44) in mol­ecule *C*, respectively. *Cg*4, *Cg*8 and *Cg*12 are the centroids of the quinoline ring systems (N4/C6–C14)in mol­ecule *A*, (N24/C26–C34) in mol­ecule *B* and (N34/C46–C54) in mol­ecule *C*, respectively.

Ring_pz_	ring_quin_	centroid–centroid	α	β	γ	inter­planar_1	inter­planar_2	offset
*Cg*1	*Cg*8^i^	3.589 (5)	2.9 (4)	9.2	8.2	3.552 (4)	3.543 (4)	0.572
*Cg*1	*Cg*12^i^	3.493 (5)	4.1 (4)	12.2	8.6	3.453 (4)	3.414 (3)	0.737
*Cg*5	*Cg*4^ii^	3.367 (5)	3.8 (4)	4.7	2.3	3.364 (4)	3.355 (4)	0.275
*Cg*5	*Cg*12^iii^	3.492 (5)	4.1 (4)	2.7	6.7	3.468 (4)	3.488 (3)	0.163
*Cg*9	*Cg*4^iv^	3.455 (6)	4.2 (4)	11.0	8.0	3.420 (4)	3.390 (4)	0.662
*Cg*9	*Cg*8^v^	3.532 (6)	2.9 (4)	3.4	5.7	3.515 (4)	3.526 (4)	0.211

Symmetry codes: (i) *x*, *y* + 1, *z* − 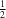; (ii) *x* + 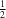, −*y* + 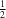, *z* + 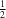; (iii) *x* − 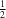, *y* − 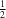, *z*; (iv) *x*, −*y* + 1, *z* + 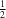; (v) *x* + 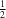, *y* + 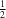, *z*.

**Table 5:** Relative percentage contributions of close contacts to the Hirshfeld surface of ligand **HL1**, and for the individual mol­ecules

Contact	**HL1**	Mol­ecule *A*	Mol­ecule *B*	Mol­ecule *C*
H⋯H	43.0	44.5	41.7	43.0
N⋯H/H⋯N	14.5	13.5	14.6	14.3
C⋯H/H⋯C	11.8	10.5	11.7	11.1
O⋯H/H⋯O	8.1	9.2	10.2	9.4
C⋯C	10.8	10.6	10.6	10.5
C⋯N	10.7	10.5	10.1	10.7

**Table 6:** Experimental details

	**HL1**	**I**
Crystal data		
Chemical formula	C_14_H_10_N_4_O	[Cu_4_(C_42_H_44_N_8_O_16_)]·2CH_4_O
*M*_r_	250.26	1235.09
Crystal system, space group	Monoclinic, *C* *c*	Triclinic, *P* 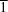
Temperature (K)	223	153
*a*, *b*, *c* (Å)	11.5047 (9), 23.410 (3), 13.4115 (11)	8.1485 (7), 11.2132 (9), 14.2662 (12)
α, β, γ (°)	90, 104.305 (8), 90	98.352 (9), 93.668 (10), 103.578 (9)
*V* (Å^3^)	3500.0 (6)	1247.11 (19)
*Z*	12	1
Radiation type	Mo *K*α	Mo *K*α
μ (mm^−1^)	0.10	1.76
Crystal size (mm)	0.50 × 0.40 × 0.30	0.50 × 0.30 × 0.25

Data collection		
Diffractometer	STOE–Siemens AED2, 4-circle	STOE *IPDS* 1
Absorption correction	–	Multi-scan (*MULABS*; Spek, 2009 ▸)
*T*_min_, *T*_max_	–	0.564, 1.000
No. of measured, independent and observed [*I* > 2σ(*I*)] reflections	4090, 4090, 2851	9825, 4499, 3906
*R*_int_	0.0	0.050
(sin θ/λ)_max_ (Å^−1^)	0.605	0.615

Refinement		
*R*[*F* ^2^ > 2σ(*F* ^2^)], *wR*(*F* ^2^), *S*	0.059, 0.134, 1.17	0.031, 0.083, 0.98
No. of reflections	4090	4499
No. of parameters	515	344
No. of restraints	2	0
H-atom treatment	H-atom parameters constrained	H atoms treated by a mixture of independent and constrained refinement
Δρ_max_, Δρ_min_ (e Å^−3^)	0.19, −0.21	0.66, −0.66

Computer programs: *STADI4*, *EXPOSE*, *CELL* and *INTEGRATE* in *IPDS-I* (Stoe & Cie, 2004 ▸), *X-RED* (Stoe & Cie, 1997 ▸), *SHELXS97* (Sheldrick, 2008 ▸), *SHELXL2018/3* (Sheldrick, 2015 ▸), *PLATON* (Spek, 2009 ▸), *Mercury* (Macrae *et al.*, 2008 ▸), *PLATON* (Spek, 2009 ▸) and *publCIF* (Westrip, 2010 ▸).

## supplementary crystallographic information

### N-(quinolin-8-yl)pyrazine-2-carboxamide (HL1) Crystal data

**Table:**

C_14_H_10_N_4_O	*F*(000) = 1560
*M**_r_* = 250.26	*D*_x_ = 1.425 Mg m^−^^3^
Monoclinic, *C**c*	Mo *K*α radiation, λ = 0.71073 Å
*a* = 11.5047 (9) Å	Cell parameters from 38 reflections
*b* = 23.410 (3) Å	θ = 10.1–19.2°
*c* = 13.4115 (11) Å	µ = 0.10 mm^−^^1^
β = 104.305 (8)°	*T* = 223 K
*V* = 3500.0 (6) Å^3^	Block, colourless
*Z* = 12	0.50 × 0.40 × 0.30 mm

### N-(quinolin-8-yl)pyrazine-2-carboxamide (HL1) Data collection

**Table:**

STOE-Siemens AED2, 4-circle diffractometer	*R*_int_ = 0.0
Radiation source: fine-focus sealed tube	θ_max_ = 25.5°, θ_min_ = 2.0°
Plane graphite monochromator	*h* = −13→13
ω/\2q scans	*k* = 0→28
4090 measured reflections	*l* = −15→16
4090 independent reflections	2 standard reflections every 60 min
2851 reflections with *I* > 2σ(*I*)	intensity decay: 1.5%

### N-(quinolin-8-yl)pyrazine-2-carboxamide (HL1) Refinement

**Table:**

Refinement on *F*^2^	Secondary atom site location: difference Fourier map
Least-squares matrix: full	Hydrogen site location: inferred from neighbouring sites
*R*[*F*^2^ > 2σ(*F*^2^)] = 0.059	H-atom parameters constrained
*wR*(*F*^2^) = 0.134	*w* = 1/[σ^2^(*F*_o_^2^) + (0.0283*P*)^2^ + 4.6085*P*] where *P* = (*F*_o_^2^ + 2*F*_c_^2^)/3
*S* = 1.17	(Δ/σ)_max_ = 0.003
4090 reflections	Δρ_max_ = 0.19 e Å^−^^3^
515 parameters	Δρ_min_ = −0.21 e Å^−^^3^
2 restraints	Extinction correction: (SHELXL-2018/3; Sheldrick, 2015), Fc^*^=kFc[1+0.001xFc^2^λ^3^/sin(2θ)]^-1/4^
Primary atom site location: structure-invariant direct methods	Extinction coefficient: 0.0021 (2)

### N-(quinolin-8-yl)pyrazine-2-carboxamide (HL1) Special details

**Table:**

Geometry. All esds (except the esd in the dihedral angle between two l.s. planes) are estimated using the full covariance matrix. The cell esds are taken into account individually in the estimation of esds in distances, angles and torsion angles; correlations between esds in cell parameters are only used when they are defined by crystal symmetry. An approximate (isotropic) treatment of cell esds is used for estimating esds involving l.s. planes.

### N-(quinolin-8-yl)pyrazine-2-carboxamide (HL1) Fractional atomic coordinates and isotropic or equivalent isotropic displacement parameters (Å^2^)

**Table:**

	*x*	*y*	*z*	*U*_iso_*/*U*_eq_	
O1	0.7337 (5)	0.0790 (3)	0.8655 (4)	0.0463 (14)	
N1	0.7597 (7)	0.1224 (4)	1.1248 (6)	0.0382 (19)	
N2	0.6261 (8)	0.2143 (4)	1.0247 (7)	0.044 (2)	
N3	0.8141 (6)	0.0339 (3)	1.0201 (5)	0.0360 (15)	
H3N	0.822207	0.037888	1.085969	0.043*	
N4	0.9191 (8)	−0.0366 (3)	1.1719 (7)	0.0331 (17)	
C1	0.7234 (8)	0.1270 (4)	1.0203 (8)	0.034 (2)	
C2	0.6608 (8)	0.1720 (4)	0.9717 (8)	0.037 (2)	
H2	0.641206	0.173510	0.899409	0.044*	
C3	0.6545 (8)	0.2114 (4)	1.1237 (8)	0.042 (2)	
H3	0.627584	0.239705	1.162515	0.050*	
C4	0.7270 (10)	0.1651 (5)	1.1748 (9)	0.045 (2)	
H4	0.752016	0.165464	1.247046	0.055*	
C5	0.7573 (6)	0.0775 (3)	0.9596 (6)	0.0320 (17)	
C6	0.8616 (9)	−0.0171 (4)	0.9887 (7)	0.031 (2)	
C7	0.8578 (10)	−0.0331 (5)	0.8907 (8)	0.042 (2)	
H7	0.818655	−0.009305	0.836235	0.050*	
C8	0.9096 (10)	−0.0833 (5)	0.8681 (9)	0.046 (3)	
H8	0.906182	−0.092985	0.799415	0.055*	
C9	0.9658 (10)	−0.1189 (5)	0.9464 (9)	0.046 (3)	
H9	1.000005	−0.153289	0.931312	0.055*	
C10	0.9724 (10)	−0.1040 (5)	1.0502 (9)	0.037 (2)	
C11	1.0315 (11)	−0.1379 (5)	1.1327 (10)	0.056 (3)	
H11	1.069668	−0.171833	1.120905	0.067*	
C12	1.0335 (10)	−0.1216 (5)	1.2294 (9)	0.046 (3)	
H12	1.073731	−0.143839	1.285804	0.055*	
C13	0.9749 (9)	−0.0713 (5)	1.2449 (8)	0.038 (2)	
H13	0.975561	−0.061557	1.313068	0.046*	
C14	0.9177 (9)	−0.0536 (4)	1.0716 (8)	0.029 (2)	
O2	1.1664 (6)	0.0867 (3)	1.2537 (5)	0.0469 (17)	
N21	1.1428 (7)	0.0431 (3)	0.9977 (6)	0.0334 (19)	
N22	1.2741 (9)	−0.0505 (4)	1.0996 (7)	0.047 (2)	
N23	1.0843 (6)	0.1305 (3)	1.1025 (5)	0.0311 (16)	
H23N	1.074944	0.126707	1.036416	0.037*	
N24	0.9780 (7)	0.2026 (4)	0.9528 (6)	0.0359 (19)	
C21	1.1773 (11)	0.0001 (5)	0.9463 (8)	0.041 (2)	
H21	1.158817	0.001603	0.874010	0.049*	
C22	1.2378 (11)	−0.0452 (5)	0.9946 (9)	0.049 (3)	
H22	1.256284	−0.074972	0.954083	0.058*	
C23	1.2382 (11)	−0.0062 (5)	1.1504 (9)	0.046 (3)	
H23	1.256952	−0.006842	1.222691	0.055*	
C24	1.1760 (8)	0.0391 (4)	1.1000 (7)	0.030 (2)	
C25	1.1415 (7)	0.0869 (4)	1.1595 (6)	0.035 (2)	
C26	1.0377 (8)	0.1811 (4)	1.1325 (7)	0.0267 (19)	
C27	1.0415 (9)	0.1959 (4)	1.2346 (7)	0.033 (2)	
H27	1.080413	0.172461	1.289764	0.040*	
C28	0.9845 (10)	0.2474 (5)	1.2516 (9)	0.045 (3)	
H28	0.987051	0.258236	1.319558	0.054*	
C29	0.9267 (11)	0.2815 (4)	1.1738 (10)	0.046 (3)	
H29	0.887722	0.314476	1.188833	0.056*	
C30	0.9238 (10)	0.2689 (5)	1.0723 (10)	0.043 (3)	
C31	0.8637 (10)	0.3020 (5)	0.9884 (9)	0.046 (3)	
H31	0.822150	0.334769	1.000800	0.056*	
C32	0.8633 (10)	0.2884 (5)	0.8881 (9)	0.051 (3)	
H32	0.824502	0.311252	0.832158	0.061*	
C33	0.9265 (10)	0.2366 (5)	0.8750 (9)	0.041 (3)	
H33	0.931273	0.226477	0.808338	0.050*	
C34	0.9778 (8)	0.2169 (4)	1.0483 (8)	0.030 (2)	
O3	1.2338 (5)	0.2464 (3)	0.8676 (4)	0.0499 (15)	
N31	1.2533 (7)	0.2882 (4)	1.1263 (6)	0.0357 (18)	
N32	1.1316 (9)	0.3839 (4)	1.0267 (8)	0.056 (2)	
N33	1.3101 (5)	0.2002 (3)	1.0212 (5)	0.0343 (14)	
H33N	1.315150	0.203851	1.086711	0.041*	
N34	1.4154 (7)	0.1285 (4)	1.1717 (6)	0.0351 (19)	
C41	1.2238 (8)	0.2920 (4)	1.0237 (7)	0.032 (2)	
C42	1.1626 (9)	0.3411 (4)	0.9757 (7)	0.040 (2)	
H42	1.143118	0.343064	0.903457	0.048*	
C43	1.1578 (10)	0.3785 (5)	1.1267 (9)	0.055 (3)	
H43	1.132327	0.406954	1.165939	0.066*	
C44	1.2214 (9)	0.3326 (4)	1.1769 (8)	0.041 (2)	
H44	1.243254	0.332380	1.249264	0.050*	
C45	1.2556 (6)	0.2439 (3)	0.9620 (6)	0.0337 (17)	
C46	1.3596 (9)	0.1501 (4)	0.9928 (8)	0.035 (2)	
C47	1.3526 (9)	0.1366 (4)	0.8925 (7)	0.036 (2)	
H47	1.310731	0.160484	0.839348	0.043*	
C48	1.4091 (10)	0.0861 (5)	0.8688 (9)	0.043 (3)	
H48	1.406998	0.077855	0.799766	0.052*	
C49	1.4661 (11)	0.0493 (5)	0.9437 (10)	0.048 (3)	
H49	1.499598	0.015108	0.926739	0.057*	
C50	1.4738 (9)	0.0637 (4)	1.0481 (9)	0.034 (2)	
C51	1.5356 (10)	0.0298 (5)	1.1340 (10)	0.052 (3)	
H51	1.576831	−0.003515	1.123746	0.062*	
C52	1.5333 (10)	0.0465 (4)	1.2297 (10)	0.048 (3)	
H52	1.573714	0.024722	1.286647	0.058*	
C53	1.4737 (10)	0.0941 (5)	1.2445 (9)	0.044 (3)	
H53	1.473984	0.103532	1.312724	0.053*	
C54	1.4176 (9)	0.1133 (5)	1.0717 (8)	0.034 (2)	

### N-(quinolin-8-yl)pyrazine-2-carboxamide (HL1) Atomic displacement parameters (Å^2^)

**Table:**

	*U*^11^	*U*^22^	*U*^33^	*U*^12^	*U*^13^	*U*^23^
O1	0.062 (4)	0.052 (4)	0.024 (3)	0.008 (3)	0.009 (3)	0.003 (2)
N1	0.038 (4)	0.041 (5)	0.033 (4)	0.001 (3)	0.004 (3)	−0.002 (3)
N2	0.047 (5)	0.031 (4)	0.052 (6)	0.005 (3)	0.011 (4)	0.010 (4)
N3	0.044 (4)	0.039 (4)	0.026 (3)	0.003 (3)	0.009 (3)	0.000 (3)
N4	0.037 (4)	0.032 (4)	0.028 (4)	0.004 (3)	0.004 (3)	0.001 (3)
C1	0.025 (4)	0.040 (5)	0.039 (5)	0.001 (3)	0.010 (4)	−0.002 (4)
C2	0.032 (5)	0.040 (5)	0.033 (5)	0.005 (4)	−0.002 (4)	0.014 (4)
C3	0.031 (5)	0.033 (5)	0.059 (7)	0.007 (4)	0.008 (4)	0.001 (4)
C4	0.043 (5)	0.037 (5)	0.048 (6)	0.005 (4)	−0.004 (4)	0.002 (4)
C5	0.030 (4)	0.028 (4)	0.040 (5)	0.001 (3)	0.013 (3)	0.000 (3)
C6	0.033 (5)	0.026 (4)	0.032 (5)	−0.007 (3)	0.004 (4)	0.001 (3)
C7	0.041 (5)	0.060 (6)	0.024 (5)	−0.007 (4)	0.008 (4)	0.010 (4)
C8	0.050 (6)	0.051 (6)	0.037 (5)	−0.008 (5)	0.015 (5)	−0.015 (5)
C9	0.057 (6)	0.042 (5)	0.043 (6)	−0.002 (5)	0.020 (5)	−0.015 (5)
C10	0.036 (5)	0.033 (5)	0.041 (6)	0.006 (4)	0.011 (4)	0.005 (4)
C11	0.058 (7)	0.043 (6)	0.075 (8)	0.017 (5)	0.031 (6)	0.013 (5)
C12	0.042 (5)	0.040 (6)	0.053 (7)	0.001 (5)	0.007 (5)	0.024 (5)
C13	0.035 (5)	0.057 (6)	0.021 (4)	−0.005 (4)	0.004 (4)	0.002 (4)
C14	0.031 (5)	0.031 (4)	0.027 (5)	−0.009 (4)	0.011 (4)	0.001 (4)
O2	0.055 (4)	0.052 (4)	0.034 (4)	0.009 (3)	0.011 (3)	0.010 (3)
N21	0.039 (4)	0.025 (4)	0.035 (5)	0.005 (3)	0.007 (4)	0.004 (3)
N22	0.059 (6)	0.038 (5)	0.043 (5)	0.009 (4)	0.011 (4)	0.010 (4)
N23	0.038 (4)	0.033 (4)	0.021 (4)	0.007 (3)	0.005 (3)	0.000 (3)
N24	0.032 (4)	0.043 (5)	0.031 (4)	−0.006 (4)	0.005 (3)	0.000 (4)
C21	0.059 (6)	0.034 (5)	0.032 (5)	−0.005 (5)	0.015 (5)	−0.005 (4)
C22	0.074 (8)	0.032 (6)	0.042 (6)	−0.005 (5)	0.019 (6)	−0.003 (5)
C23	0.054 (6)	0.042 (6)	0.045 (7)	−0.005 (5)	0.020 (5)	0.010 (5)
C24	0.033 (5)	0.027 (5)	0.029 (5)	−0.008 (4)	0.006 (4)	0.004 (4)
C25	0.025 (4)	0.055 (6)	0.022 (4)	−0.004 (4)	−0.001 (3)	0.016 (4)
C26	0.020 (4)	0.033 (5)	0.027 (5)	−0.002 (4)	0.004 (3)	0.004 (4)
C27	0.039 (5)	0.029 (5)	0.031 (5)	−0.014 (4)	0.010 (4)	−0.009 (4)
C28	0.053 (7)	0.044 (6)	0.046 (6)	−0.015 (5)	0.025 (5)	−0.009 (5)
C29	0.054 (6)	0.025 (5)	0.068 (8)	−0.004 (4)	0.031 (6)	−0.008 (5)
C30	0.038 (6)	0.041 (6)	0.054 (7)	−0.005 (5)	0.019 (5)	−0.006 (5)
C31	0.050 (6)	0.034 (5)	0.055 (7)	−0.002 (5)	0.015 (5)	0.002 (5)
C32	0.043 (6)	0.058 (8)	0.049 (7)	−0.001 (6)	0.006 (5)	−0.005 (5)
C33	0.043 (6)	0.036 (6)	0.042 (6)	0.001 (4)	0.006 (5)	0.013 (5)
C34	0.022 (4)	0.025 (4)	0.038 (5)	−0.003 (3)	0.001 (4)	−0.009 (4)
O3	0.062 (4)	0.064 (4)	0.024 (3)	0.007 (3)	0.011 (3)	0.009 (3)
N31	0.036 (4)	0.038 (4)	0.032 (4)	−0.001 (3)	0.005 (3)	0.002 (3)
N32	0.061 (5)	0.046 (5)	0.061 (6)	0.009 (4)	0.015 (5)	0.010 (5)
N33	0.035 (3)	0.043 (4)	0.026 (3)	0.000 (3)	0.008 (3)	0.002 (3)
N34	0.038 (4)	0.039 (4)	0.028 (4)	−0.007 (3)	0.008 (3)	0.000 (3)
C41	0.026 (4)	0.035 (5)	0.036 (5)	−0.002 (3)	0.008 (4)	0.009 (4)
C42	0.041 (5)	0.043 (6)	0.033 (5)	0.007 (4)	0.006 (4)	0.008 (4)
C43	0.054 (6)	0.053 (7)	0.056 (7)	0.012 (5)	0.009 (5)	0.009 (5)
C44	0.047 (5)	0.040 (5)	0.038 (5)	0.012 (4)	0.013 (4)	−0.001 (4)
C45	0.038 (4)	0.026 (4)	0.036 (4)	0.001 (3)	0.007 (3)	0.003 (3)
C46	0.039 (5)	0.033 (5)	0.038 (5)	−0.006 (4)	0.018 (4)	−0.013 (4)
C47	0.039 (5)	0.042 (5)	0.030 (5)	−0.005 (4)	0.014 (4)	−0.003 (4)
C48	0.045 (6)	0.047 (6)	0.045 (6)	−0.017 (5)	0.024 (5)	−0.021 (5)
C49	0.050 (6)	0.043 (6)	0.056 (7)	−0.008 (5)	0.024 (5)	−0.023 (5)
C50	0.036 (5)	0.024 (5)	0.045 (6)	−0.008 (4)	0.015 (4)	0.000 (4)
C51	0.044 (5)	0.037 (5)	0.082 (9)	0.005 (4)	0.032 (6)	0.013 (5)
C52	0.039 (5)	0.042 (6)	0.063 (7)	0.010 (4)	0.012 (5)	0.036 (5)
C53	0.037 (5)	0.056 (6)	0.035 (5)	−0.009 (4)	0.002 (4)	0.001 (4)
C54	0.035 (5)	0.040 (5)	0.027 (5)	−0.007 (4)	0.013 (4)	−0.001 (4)

### N-(quinolin-8-yl)pyrazine-2-carboxamide (HL1) Geometric parameters (Å, º)

**Table:**

O1—C5	1.224 (9)	C26—C27	1.403 (13)
N1—C4	1.311 (14)	C26—C34	1.439 (14)
N1—C1	1.364 (12)	C27—C28	1.418 (15)
N2—C3	1.289 (13)	C27—H27	0.9400
N2—C2	1.337 (13)	C28—C29	1.351 (17)
N3—C5	1.366 (10)	C28—H28	0.9400
N3—C6	1.419 (11)	C29—C30	1.385 (17)
N3—H3N	0.8700	C29—H29	0.9400
N4—C13	1.312 (14)	C30—C31	1.400 (16)
N4—C14	1.398 (13)	C30—C34	1.438 (15)
C1—C2	1.349 (14)	C31—C32	1.380 (16)
C1—C5	1.521 (11)	C31—H31	0.9400
C2—H2	0.9400	C32—C33	1.447 (16)
C3—C4	1.435 (15)	C32—H32	0.9400
C3—H3	0.9400	C33—H33	0.9400
C4—H4	0.9400	O3—C45	1.230 (9)
C6—C7	1.358 (13)	N31—C41	1.337 (12)
C6—C14	1.424 (14)	N31—C44	1.340 (12)
C7—C8	1.385 (15)	N32—C43	1.306 (14)
C7—H7	0.9400	N32—C42	1.312 (13)
C8—C9	1.371 (17)	N33—C45	1.350 (10)
C8—H8	0.9400	N33—C46	1.399 (11)
C9—C10	1.419 (16)	N33—H33N	0.8700
C9—H9	0.9400	N34—C53	1.314 (14)
C10—C11	1.395 (16)	N34—C54	1.395 (13)
C10—C14	1.400 (11)	C41—C42	1.417 (14)
C11—C12	1.347 (17)	C41—C45	1.494 (12)
C11—H11	0.9400	C42—H42	0.9400
C12—C13	1.398 (15)	C43—C44	1.378 (16)
C12—H12	0.9400	C43—H43	0.9400
C13—H13	0.9400	C44—H44	0.9400
O2—C25	1.224 (11)	C46—C47	1.366 (13)
N21—C24	1.333 (12)	C46—C54	1.398 (15)
N21—C21	1.334 (13)	C47—C48	1.422 (13)
N22—C23	1.360 (15)	C47—H47	0.9400
N22—C22	1.372 (15)	C48—C49	1.362 (17)
N23—C25	1.347 (11)	C48—H48	0.9400
N23—C26	1.399 (11)	C49—C50	1.421 (16)
N23—H23N	0.8700	C49—H49	0.9400
N24—C34	1.324 (13)	C50—C54	1.403 (11)
N24—C33	1.329 (13)	C50—C51	1.435 (16)
C21—C22	1.344 (17)	C51—C52	1.348 (16)
C21—H21	0.9400	C51—H51	0.9400
C22—H22	0.9400	C52—C53	1.350 (15)
C23—C24	1.362 (16)	C52—H52	0.9400
C23—H23	0.9400	C53—H53	0.9400
C24—C25	1.484 (13)		
			
C4—N1—C1	114.3 (10)	C26—C27—C28	117.5 (10)
C3—N2—C2	118.4 (9)	C26—C27—H27	121.2
C5—N3—C6	128.0 (7)	C28—C27—H27	121.2
C5—N3—H3N	116.0	C29—C28—C27	122.5 (11)
C6—N3—H3N	116.0	C29—C28—H28	118.7
C13—N4—C14	115.5 (8)	C27—C28—H28	118.7
C2—C1—N1	123.4 (9)	C28—C29—C30	121.4 (10)
C2—C1—C5	120.8 (9)	C28—C29—H29	119.3
N1—C1—C5	115.8 (9)	C30—C29—H29	119.3
N2—C2—C1	121.0 (9)	C29—C30—C31	124.0 (11)
N2—C2—H2	119.5	C29—C30—C34	119.5 (12)
C1—C2—H2	119.5	C31—C30—C34	116.3 (11)
N2—C3—C4	120.1 (9)	C32—C31—C30	122.5 (11)
N2—C3—H3	120.0	C32—C31—H31	118.8
C4—C3—H3	120.0	C30—C31—H31	118.8
N1—C4—C3	122.6 (10)	C31—C32—C33	115.5 (11)
N1—C4—H4	118.7	C31—C32—H32	122.2
C3—C4—H4	118.7	C33—C32—H32	122.2
O1—C5—N3	125.8 (7)	N24—C33—C32	123.1 (11)
O1—C5—C1	120.6 (8)	N24—C33—H33	118.4
N3—C5—C1	113.5 (7)	C32—C33—H33	118.4
C7—C6—N3	126.7 (9)	N24—C34—C30	122.3 (11)
C7—C6—C14	119.3 (9)	N24—C34—C26	119.8 (8)
N3—C6—C14	114.0 (8)	C30—C34—C26	117.9 (10)
C6—C7—C8	122.3 (10)	C41—N31—C44	116.1 (9)
C6—C7—H7	118.9	C43—N32—C42	116.0 (10)
C8—C7—H7	118.9	C45—N33—C46	129.6 (7)
C9—C8—C7	119.7 (10)	C45—N33—H33N	115.2
C9—C8—H8	120.2	C46—N33—H33N	115.2
C7—C8—H8	120.2	C53—N34—C54	115.5 (9)
C8—C9—C10	120.2 (10)	N31—C41—C42	119.5 (9)
C8—C9—H9	119.9	N31—C41—C45	119.1 (8)
C10—C9—H9	119.9	C42—C41—C45	121.4 (8)
C11—C10—C14	118.2 (9)	N32—C42—C41	123.5 (9)
C11—C10—C9	122.4 (10)	N32—C42—H42	118.3
C14—C10—C9	119.4 (9)	C41—C42—H42	118.3
C12—C11—C10	119.5 (10)	N32—C43—C44	122.5 (11)
C12—C11—H11	120.3	N32—C43—H43	118.7
C10—C11—H11	120.3	C44—C43—H43	118.7
C11—C12—C13	119.2 (10)	N31—C44—C43	122.3 (10)
C11—C12—H12	120.4	N31—C44—H44	118.8
C13—C12—H12	120.4	C43—C44—H44	118.8
N4—C13—C12	125.1 (10)	O3—C45—N33	126.1 (7)
N4—C13—H13	117.4	O3—C45—C41	121.2 (8)
C12—C13—H13	117.4	N33—C45—C41	112.7 (7)
N4—C14—C10	122.5 (8)	C47—C46—C54	120.3 (9)
N4—C14—C6	118.4 (9)	C47—C46—N33	122.3 (9)
C10—C14—C6	119.1 (8)	C54—C46—N33	117.5 (8)
C24—N21—C21	115.7 (9)	C46—C47—C48	119.6 (10)
C23—N22—C22	113.1 (10)	C46—C47—H47	120.2
C25—N23—C26	130.3 (8)	C48—C47—H47	120.2
C25—N23—H23N	114.9	C49—C48—C47	121.6 (10)
C26—N23—H23N	114.9	C49—C48—H48	119.2
C34—N24—C33	120.0 (9)	C47—C48—H48	119.2
N21—C21—C22	122.0 (11)	C48—C49—C50	118.6 (10)
N21—C21—H21	119.0	C48—C49—H49	120.7
C22—C21—H21	119.0	C50—C49—H49	120.7
C21—C22—N22	123.8 (11)	C54—C50—C49	119.9 (9)
C21—C22—H22	118.1	C54—C50—C51	116.2 (8)
N22—C22—H22	118.1	C49—C50—C51	123.9 (10)
N22—C23—C24	122.1 (11)	C52—C51—C50	118.8 (10)
N22—C23—H23	118.9	C52—C51—H51	120.6
C24—C23—H23	118.9	C50—C51—H51	120.6
N21—C24—C23	123.2 (9)	C51—C52—C53	120.7 (10)
N21—C24—C25	117.0 (8)	C51—C52—H52	119.7
C23—C24—C25	119.8 (9)	C53—C52—H52	119.7
O2—C25—N23	123.1 (9)	N34—C53—C52	125.5 (11)
O2—C25—C24	121.8 (9)	N34—C53—H53	117.2
N23—C25—C24	115.1 (8)	C52—C53—H53	117.2
N23—C26—C27	124.6 (9)	N34—C54—C46	116.9 (9)
N23—C26—C34	114.2 (8)	N34—C54—C50	123.2 (8)
C27—C26—C34	121.1 (9)	C46—C54—C50	120.0 (7)
			
C4—N1—C1—C2	1.0 (13)	C27—C28—C29—C30	−2.4 (17)
C4—N1—C1—C5	−178.7 (8)	C28—C29—C30—C31	178.9 (11)
C3—N2—C2—C1	0.3 (14)	C28—C29—C30—C34	3.3 (17)
N1—C1—C2—N2	−2.7 (14)	C29—C30—C31—C32	179.5 (11)
C5—C1—C2—N2	177.0 (8)	C34—C30—C31—C32	−4.8 (16)
C2—N2—C3—C4	3.3 (14)	C30—C31—C32—C33	1.6 (16)
C1—N1—C4—C3	2.7 (14)	C34—N24—C33—C32	−3.1 (16)
N2—C3—C4—N1	−5.1 (16)	C31—C32—C33—N24	2.6 (16)
C6—N3—C5—O1	2.7 (13)	C33—N24—C34—C30	−0.4 (15)
C6—N3—C5—C1	−177.2 (8)	C33—N24—C34—C26	−177.6 (9)
C2—C1—C5—O1	3.8 (12)	C29—C30—C34—N24	−179.8 (8)
N1—C1—C5—O1	−176.5 (8)	C31—C30—C34—N24	4.3 (16)
C2—C1—C5—N3	−176.2 (8)	C29—C30—C34—C26	−2.6 (16)
N1—C1—C5—N3	3.4 (10)	C31—C30—C34—C26	−178.5 (7)
C5—N3—C6—C7	−1.3 (14)	N23—C26—C34—N24	−4.6 (12)
C5—N3—C6—C14	178.9 (7)	C27—C26—C34—N24	178.4 (9)
N3—C6—C7—C8	178.4 (9)	N23—C26—C34—C30	178.1 (9)
C14—C6—C7—C8	−1.8 (15)	C27—C26—C34—C30	1.1 (14)
C6—C7—C8—C9	0.8 (17)	C44—N31—C41—C42	0.0 (12)
C7—C8—C9—C10	−0.9 (17)	C44—N31—C41—C45	−179.6 (8)
C8—C9—C10—C11	−178.2 (11)	C43—N32—C42—C41	−1.7 (15)
C8—C9—C10—C14	2.1 (15)	N31—C41—C42—N32	−0.5 (14)
C14—C10—C11—C12	0.5 (15)	C45—C41—C42—N32	179.1 (9)
C9—C10—C11—C12	−179.3 (11)	C42—N32—C43—C44	4.3 (17)
C10—C11—C12—C13	0.7 (17)	C41—N31—C44—C43	2.5 (14)
C14—N4—C13—C12	1.7 (15)	N32—C43—C44—N31	−5.0 (18)
C11—C12—C13—N4	−1.9 (18)	C46—N33—C45—O3	3.7 (13)
C13—N4—C14—C10	−0.3 (12)	C46—N33—C45—C41	−175.4 (8)
C13—N4—C14—C6	−178.2 (9)	N31—C41—C45—O3	−177.5 (8)
C11—C10—C14—N4	−0.7 (12)	C42—C41—C45—O3	2.9 (12)
C9—C10—C14—N4	179.0 (11)	N31—C41—C45—N33	1.6 (10)
C11—C10—C14—C6	177.2 (11)	C42—C41—C45—N33	−178.0 (8)
C9—C10—C14—C6	−3.1 (12)	C45—N33—C46—C47	−3.9 (14)
C7—C6—C14—N4	−179.1 (9)	C45—N33—C46—C54	176.2 (7)
N3—C6—C14—N4	0.8 (12)	C54—C46—C47—C48	−2.6 (14)
C7—C6—C14—C10	2.9 (12)	N33—C46—C47—C48	177.4 (9)
N3—C6—C14—C10	−177.2 (7)	C46—C47—C48—C49	2.8 (15)
C24—N21—C21—C22	2.4 (16)	C47—C48—C49—C50	−3.2 (16)
N21—C21—C22—N22	−3.1 (19)	C48—C49—C50—C54	3.5 (14)
C23—N22—C22—C21	2.7 (17)	C48—C49—C50—C51	−177.4 (10)
C22—N22—C23—C24	−2.0 (16)	C54—C50—C51—C52	1.6 (13)
C21—N21—C24—C23	−1.8 (15)	C49—C50—C51—C52	−177.6 (10)
C21—N21—C24—C25	178.6 (9)	C50—C51—C52—C53	0.3 (16)
N22—C23—C24—N21	1.8 (16)	C54—N34—C53—C52	−0.3 (15)
N22—C23—C24—C25	−178.6 (9)	C51—C52—C53—N34	−1.0 (18)
C26—N23—C25—O2	−3.2 (15)	C53—N34—C54—C46	−178.0 (9)
C26—N23—C25—C24	178.6 (8)	C53—N34—C54—C50	2.4 (12)
N21—C24—C25—O2	178.9 (9)	C47—C46—C54—N34	−176.6 (9)
C23—C24—C25—O2	−0.7 (13)	N33—C46—C54—N34	3.4 (13)
N21—C24—C25—N23	−2.8 (11)	C47—C46—C54—C50	3.0 (13)
C23—C24—C25—N23	177.5 (9)	N33—C46—C54—C50	−177.0 (7)
C25—N23—C26—C27	−0.7 (15)	C49—C50—C54—N34	176.2 (11)
C25—N23—C26—C34	−177.6 (8)	C51—C50—C54—N34	−3.0 (11)
N23—C26—C27—C28	−176.8 (8)	C49—C50—C54—C46	−3.4 (11)
C34—C26—C27—C28	−0.2 (14)	C51—C50—C54—C46	177.4 (11)
C26—C27—C28—C29	0.8 (16)		

### N-(quinolin-8-yl)pyrazine-2-carboxamide (HL1) Hydrogen-bond geometry (Å, º)

**Table:**

*D*—H···*A*	*D*—H	H···*A*	*D*···*A*	*D*—H···*A*
N3—H3*N*···N1	0.87	2.21	2.661 (10)	112
N3—H3*N*···N4	0.87	2.23	2.667 (10)	111
C7—H7···O1	0.94	2.36	2.967 (13)	122
N23—H23*N*···N21	0.87	2.22	2.662 (10)	112
N23—H23*N*···N24	0.87	2.24	2.675 (11)	110
C21—H21···O2^i^	0.94	2.64	3.264 (12)	125
C22—H22···O2^i^	0.94	2.64	3.278 (13)	125
C27—H27···O2	0.94	2.34	2.912 (13)	119
N33—H33*N*···N31	0.87	2.21	2.667 (10)	113
N33—H33*N*···N34	0.87	2.26	2.674 (11)	109
C44—H44···O1^ii^	0.94	2.61	3.244 (11)	125
C47—H47···O3	0.94	2.27	2.893 (12)	123

Symmetry codes: (i) *x*, −*y*, *z*−1/2; (ii) *x*+1/2, −*y*+1/2, *z*+1/2.

### Hexa-µ-acetato-1:2κ2O:O';2:3κ8O:O'; 3:4κ2O:O'-dimethanol-1κO,2κO-bis[N-(quinolin-8-yl)pyrazine-2-carboxamide]-1κ3N,N',N''; 4κ3N,N',N''-tetracopper(II) methanol disolvate (I) Crystal data

**Table:**

[Cu_4_(C_42_H_44_N_8_O_16_)]·2CH_4_O	*Z* = 1
*M**_r_* = 1235.09	*F*(000) = 632
Triclinic, *P*1	*D*_x_ = 1.645 Mg m^−^^3^
*a* = 8.1485 (7) Å	Mo *K*α radiation, λ = 0.71073 Å
*b* = 11.2132 (9) Å	Cell parameters from 8000 reflections
*c* = 14.2662 (12) Å	θ = 2.2–25.9°
α = 98.352 (9)°	µ = 1.76 mm^−^^1^
β = 93.668 (10)°	*T* = 153 K
γ = 103.578 (9)°	Block, green
*V* = 1247.11 (19) Å^3^	0.50 × 0.30 × 0.25 mm

### Hexa-µ-acetato-1:2κ2O:O';2:3κ8O:O'; 3:4κ2O:O'-dimethanol-1κO,2κO-bis[N-(quinolin-8-yl)pyrazine-2-carboxamide]-1κ3N,N',N''; 4κ3N,N',N''-tetracopper(II) methanol disolvate (I) Data collection

**Table:**

STOE IPDS 1 diffractometer	4499 independent reflections
Radiation source: fine-focus sealed tube	3906 reflections with *I* > 2σ(*I*)
Plane graphite monochromator	*R*_int_ = 0.050
φ rotation scans	θ_max_ = 25.9°, θ_min_ = 2.2°
Absorption correction: multi-scan (MULABS; Spek, 2009)	*h* = −10→10
*T*_min_ = 0.564, *T*_max_ = 1.000	*k* = −13→13
9825 measured reflections	*l* = −17→17

### Hexa-µ-acetato-1:2κ2O:O';2:3κ8O:O'; 3:4κ2O:O'-dimethanol-1κO,2κO-bis[N-(quinolin-8-yl)pyrazine-2-carboxamide]-1κ3N,N',N''; 4κ3N,N',N''-tetracopper(II) methanol disolvate (I) Refinement

**Table:**

Refinement on *F*^2^	Primary atom site location: structure-invariant direct methods
Least-squares matrix: full	Secondary atom site location: difference Fourier map
*R*[*F*^2^ > 2σ(*F*^2^)] = 0.031	Hydrogen site location: mixed
*wR*(*F*^2^) = 0.083	H atoms treated by a mixture of independent and constrained refinement
*S* = 0.98	*w* = 1/[σ^2^(*F*_o_^2^) + (0.0579*P*)^2^] where *P* = (*F*_o_^2^ + 2*F*_c_^2^)/3
4499 reflections	(Δ/σ)_max_ = 0.001
344 parameters	Δρ_max_ = 0.66 e Å^−^^3^
0 restraints	Δρ_min_ = −0.66 e Å^−^^3^

### Hexa-µ-acetato-1:2κ2O:O';2:3κ8O:O'; 3:4κ2O:O'-dimethanol-1κO,2κO-bis[N-(quinolin-8-yl)pyrazine-2-carboxamide]-1κ3N,N',N''; 4κ3N,N',N''-tetracopper(II) methanol disolvate (I) Special details

**Table:**

Geometry. All esds (except the esd in the dihedral angle between two l.s. planes) are estimated using the full covariance matrix. The cell esds are taken into account individually in the estimation of esds in distances, angles and torsion angles; correlations between esds in cell parameters are only used when they are defined by crystal symmetry. An approximate (isotropic) treatment of cell esds is used for estimating esds involving l.s. planes.

### Hexa-µ-acetato-1:2κ2O:O';2:3κ8O:O'; 3:4κ2O:O'-dimethanol-1κO,2κO-bis[N-(quinolin-8-yl)pyrazine-2-carboxamide]-1κ3N,N',N''; 4κ3N,N',N''-tetracopper(II) methanol disolvate (I) Fractional atomic coordinates and isotropic or equivalent isotropic displacement parameters (Å^2^)

**Table:**

	*x*	*y*	*z*	*U*_iso_*/*U*_eq_	
Cu1	0.39598 (3)	0.31687 (2)	0.17585 (2)	0.01373 (9)	
O2	0.1648 (2)	0.37443 (17)	0.10360 (12)	0.0196 (4)	
H2O	0.208 (3)	0.440 (3)	0.081 (2)	0.020 (7)*	
C15	0.0276 (3)	0.3951 (3)	0.15486 (19)	0.0295 (6)	
H15C	−0.070186	0.391617	0.109933	0.044*	
H15B	0.061424	0.477100	0.195227	0.044*	
H15A	−0.003095	0.330750	0.194798	0.044*	
N1	0.5500 (2)	0.49209 (19)	0.19753 (13)	0.0158 (4)	
N2	0.7940 (2)	0.7145 (2)	0.20796 (15)	0.0220 (4)	
N3	0.5020 (2)	0.30915 (19)	0.05748 (13)	0.0143 (4)	
N4	0.2904 (2)	0.13793 (19)	0.12373 (13)	0.0151 (4)	
O1	0.6953 (2)	0.42282 (17)	−0.02929 (12)	0.0214 (4)	
C1	0.6469 (3)	0.5128 (2)	0.12584 (15)	0.0145 (4)	
C2	0.7678 (3)	0.6243 (2)	0.13166 (17)	0.0193 (5)	
H2	0.834295	0.637233	0.080020	0.023*	
C3	0.6944 (3)	0.6927 (2)	0.27740 (17)	0.0221 (5)	
H3	0.707511	0.755074	0.331961	0.027*	
C4	0.5719 (3)	0.5817 (2)	0.27251 (16)	0.0193 (5)	
H4	0.503163	0.569718	0.323337	0.023*	
C5	0.6164 (3)	0.4088 (2)	0.04218 (15)	0.0145 (5)	
C6	0.4453 (3)	0.1982 (2)	−0.00583 (15)	0.0142 (4)	
C7	0.4895 (3)	0.1676 (2)	−0.09636 (15)	0.0174 (5)	
H7	0.568342	0.227202	−0.122700	0.021*	
C8	0.4187 (3)	0.0487 (2)	−0.14995 (16)	0.0209 (5)	
H8	0.450487	0.029735	−0.212245	0.025*	
C9	0.3049 (3)	−0.0406 (2)	−0.11458 (16)	0.0209 (5)	
H9	0.258555	−0.120132	−0.152212	0.025*	
C10	0.2569 (3)	−0.0133 (2)	−0.02131 (16)	0.0172 (5)	
C11	0.1416 (3)	−0.0996 (2)	0.02149 (18)	0.0212 (5)	
H11	0.088973	−0.180113	−0.012911	0.025*	
C12	0.1067 (3)	−0.0660 (2)	0.11283 (17)	0.0221 (5)	
H12	0.030098	−0.123070	0.142415	0.027*	
C13	0.1849 (3)	0.0530 (2)	0.16210 (16)	0.0183 (5)	
H13	0.161656	0.074312	0.225945	0.022*	
C14	0.3277 (3)	0.1054 (2)	0.03234 (15)	0.0141 (4)	
Cu2	0.44706 (3)	0.08030 (3)	0.45535 (2)	0.01311 (9)	
O3	0.3072 (2)	0.34649 (16)	0.29775 (11)	0.0178 (3)	
O4	0.3848 (2)	0.19608 (16)	0.36021 (11)	0.0195 (4)	
O5	0.5820 (2)	0.21036 (16)	0.55744 (12)	0.0220 (4)	
O6	0.6575 (2)	0.08152 (17)	0.39303 (11)	0.0208 (4)	
O7	0.3245 (2)	−0.07186 (17)	0.36824 (12)	0.0250 (4)	
O8	0.25541 (19)	0.05939 (17)	0.53366 (12)	0.0214 (4)	
C16	0.3260 (3)	0.2889 (2)	0.36657 (15)	0.0144 (4)	
C17	0.2738 (4)	0.3410 (3)	0.46051 (17)	0.0300 (6)	
H17A	0.225199	0.411380	0.452304	0.045*	
H17B	0.373388	0.369265	0.507758	0.045*	
H17C	0.188966	0.276304	0.482413	0.045*	
C18	0.6718 (3)	0.1805 (2)	0.62131 (16)	0.0179 (5)	
C19	0.7880 (3)	0.2856 (3)	0.69104 (19)	0.0294 (6)	
H19A	0.736862	0.295786	0.751097	0.044*	
H19B	0.804464	0.362812	0.664396	0.044*	
H19C	0.897884	0.266140	0.702785	0.044*	
C20	0.7617 (3)	0.0184 (2)	0.40933 (15)	0.0159 (5)	
C21	0.9158 (3)	0.0332 (3)	0.35565 (17)	0.0227 (5)	
H21C	1.015191	0.034582	0.398686	0.034*	
H21B	0.934162	0.111348	0.330169	0.034*	
H21A	0.898758	−0.036643	0.303063	0.034*	
O9	0.7083 (3)	0.2935 (2)	0.28288 (15)	0.0402 (5)	
H9O	0.646942	0.230274	0.299405	0.060*	
C22	0.7910 (4)	0.3767 (3)	0.3638 (2)	0.0457 (8)	
H22A	0.853986	0.453730	0.344812	0.069*	
H22B	0.869998	0.339429	0.397142	0.069*	
H22C	0.707048	0.395299	0.406157	0.069*	

### Hexa-µ-acetato-1:2κ2O:O';2:3κ8O:O'; 3:4κ2O:O'-dimethanol-1κO,2κO-bis[N-(quinolin-8-yl)pyrazine-2-carboxamide]-1κ3N,N',N''; 4κ3N,N',N''-tetracopper(II) methanol disolvate (I) Atomic displacement parameters (Å^2^)

**Table:**

	*U*^11^	*U*^22^	*U*^33^	*U*^12^	*U*^13^	*U*^23^
Cu1	0.01849 (15)	0.01031 (18)	0.01175 (14)	0.00192 (11)	0.00404 (10)	0.00134 (11)
O2	0.0199 (8)	0.0199 (11)	0.0213 (8)	0.0052 (7)	0.0047 (6)	0.0088 (8)
C15	0.0202 (12)	0.0357 (18)	0.0291 (13)	0.0058 (11)	0.0026 (10)	−0.0044 (12)
N1	0.0172 (9)	0.0165 (12)	0.0143 (9)	0.0048 (7)	0.0002 (7)	0.0042 (8)
N2	0.0228 (10)	0.0146 (12)	0.0249 (10)	−0.0001 (8)	−0.0024 (8)	0.0016 (9)
N3	0.0158 (8)	0.0126 (11)	0.0137 (9)	0.0013 (7)	0.0013 (7)	0.0028 (8)
N4	0.0155 (8)	0.0142 (11)	0.0158 (9)	0.0044 (7)	0.0020 (7)	0.0021 (8)
O1	0.0257 (8)	0.0193 (10)	0.0188 (8)	0.0015 (7)	0.0093 (7)	0.0052 (7)
C1	0.0143 (10)	0.0146 (13)	0.0165 (10)	0.0061 (8)	−0.0003 (8)	0.0055 (9)
C2	0.0171 (10)	0.0168 (14)	0.0228 (11)	0.0019 (9)	0.0007 (9)	0.0040 (10)
C3	0.0273 (12)	0.0150 (14)	0.0212 (12)	0.0032 (9)	−0.0031 (9)	−0.0008 (10)
C4	0.0238 (11)	0.0187 (14)	0.0151 (10)	0.0059 (9)	0.0003 (9)	0.0016 (9)
C5	0.0141 (10)	0.0141 (14)	0.0163 (10)	0.0049 (8)	−0.0006 (8)	0.0044 (9)
C6	0.0149 (9)	0.0134 (13)	0.0151 (10)	0.0055 (8)	−0.0014 (8)	0.0029 (9)
C7	0.0208 (10)	0.0206 (14)	0.0123 (10)	0.0068 (9)	0.0020 (8)	0.0045 (9)
C8	0.0265 (12)	0.0248 (15)	0.0126 (10)	0.0108 (10)	0.0014 (9)	0.0002 (10)
C9	0.0277 (12)	0.0164 (14)	0.0162 (11)	0.0061 (9)	−0.0031 (9)	−0.0036 (9)
C10	0.0174 (10)	0.0158 (14)	0.0172 (11)	0.0047 (9)	−0.0030 (8)	0.0002 (9)
C11	0.0209 (11)	0.0125 (14)	0.0264 (12)	0.0009 (9)	−0.0027 (9)	−0.0015 (10)
C12	0.0198 (11)	0.0177 (15)	0.0258 (12)	−0.0026 (9)	0.0035 (9)	0.0049 (10)
C13	0.0201 (11)	0.0165 (14)	0.0176 (11)	0.0021 (9)	0.0050 (8)	0.0034 (9)
C14	0.0149 (9)	0.0147 (13)	0.0135 (10)	0.0057 (8)	−0.0001 (8)	0.0019 (9)
Cu2	0.01474 (14)	0.01284 (18)	0.01143 (14)	0.00311 (10)	0.00007 (10)	0.00198 (11)
O3	0.0262 (8)	0.0157 (10)	0.0141 (7)	0.0083 (6)	0.0048 (6)	0.0043 (7)
O4	0.0279 (8)	0.0187 (10)	0.0140 (7)	0.0094 (7)	0.0004 (6)	0.0046 (7)
O5	0.0240 (8)	0.0170 (10)	0.0218 (8)	0.0028 (7)	−0.0047 (7)	−0.0003 (7)
O6	0.0231 (8)	0.0236 (11)	0.0198 (8)	0.0098 (7)	0.0079 (6)	0.0081 (7)
O7	0.0314 (9)	0.0198 (11)	0.0204 (8)	0.0039 (7)	−0.0073 (7)	0.0006 (7)
O8	0.0191 (8)	0.0252 (11)	0.0234 (8)	0.0077 (7)	0.0059 (6)	0.0103 (8)
C16	0.0140 (10)	0.0149 (14)	0.0133 (10)	0.0022 (8)	−0.0002 (8)	0.0026 (9)
C17	0.0464 (15)	0.0338 (18)	0.0164 (12)	0.0196 (13)	0.0102 (11)	0.0066 (11)
C18	0.0141 (10)	0.0203 (15)	0.0165 (11)	0.0023 (9)	0.0021 (8)	−0.0028 (9)
C19	0.0265 (12)	0.0227 (17)	0.0313 (14)	0.0005 (10)	−0.0082 (11)	−0.0064 (11)
C20	0.0174 (10)	0.0140 (14)	0.0131 (10)	0.0011 (8)	−0.0002 (8)	−0.0030 (9)
C21	0.0198 (11)	0.0244 (16)	0.0234 (12)	0.0041 (9)	0.0048 (9)	0.0037 (10)
O9	0.0560 (13)	0.0284 (14)	0.0405 (11)	0.0107 (10)	0.0252 (10)	0.0108 (10)
C22	0.0507 (18)	0.033 (2)	0.053 (2)	0.0066 (14)	0.0113 (15)	0.0083 (16)

### Hexa-µ-acetato-1:2κ2O:O';2:3κ8O:O'; 3:4κ2O:O'-dimethanol-1κO,2κO-bis[N-(quinolin-8-yl)pyrazine-2-carboxamide]-1κ3N,N',N''; 4κ3N,N',N''-tetracopper(II) methanol disolvate (I) Geometric parameters (Å, º)

**Table:**

Cu1—N1	2.037 (2)	C11—C12	1.370 (4)
Cu1—N3	1.9457 (18)	C11—H11	0.9500
Cu1—N4	1.998 (2)	C12—C13	1.397 (4)
Cu1—O2	2.3541 (16)	C12—H12	0.9500
Cu1—O3	1.9401 (15)	C13—H13	0.9500
O2—C15	1.420 (3)	Cu2—Cu2^i^	2.6202 (6)
O2—H2O	0.85 (3)	Cu2—O4	2.1255 (16)
C15—H15C	0.9800	Cu2—O5	1.9703 (17)
C15—H15B	0.9800	Cu2—O6	1.9793 (15)
C15—H15A	0.9800	Cu2—O7	1.9692 (18)
N1—C4	1.328 (3)	Cu2—O8	1.9671 (16)
N1—C1	1.345 (3)	O3—C16	1.271 (3)
N2—C3	1.334 (3)	O4—C16	1.238 (3)
N2—C2	1.343 (3)	O5—C18	1.264 (3)
N3—C5	1.331 (3)	O6—C20	1.258 (3)
N3—C6	1.388 (3)	O7—C18^i^	1.256 (3)
N4—C13	1.332 (3)	O8—C20^i^	1.267 (3)
N4—C14	1.375 (3)	C16—C17	1.510 (3)
O1—C5	1.249 (3)	C17—H17A	0.9800
C1—C2	1.386 (3)	C17—H17B	0.9800
C1—C5	1.506 (3)	C17—H17C	0.9800
C2—H2	0.9500	C18—C19	1.513 (3)
C3—C4	1.391 (3)	C19—H19A	0.9800
C3—H3	0.9500	C19—H19B	0.9800
C4—H4	0.9500	C19—H19C	0.9800
C6—C7	1.380 (3)	C20—C21	1.501 (3)
C6—C14	1.436 (3)	C21—H21C	0.9800
C7—C8	1.407 (4)	C21—H21B	0.9800
C7—H7	0.9500	C21—H21A	0.9800
C8—C9	1.375 (4)	O9—C22	1.396 (4)
C8—H8	0.9500	O9—H9O	0.8400
C9—C10	1.422 (3)	C22—H22A	0.9800
C9—H9	0.9500	C22—H22B	0.9800
C10—C14	1.406 (3)	C22—H22C	0.9800
C10—C11	1.418 (3)		
			
O3—Cu1—N3	172.66 (8)	C11—C12—H12	120.3
O3—Cu1—N4	105.05 (7)	C13—C12—H12	120.3
N3—Cu1—N4	82.29 (8)	N4—C13—C12	123.0 (2)
O3—Cu1—N1	91.72 (7)	N4—C13—H13	118.5
N3—Cu1—N1	80.94 (8)	C12—C13—H13	118.5
N4—Cu1—N1	162.67 (8)	N4—C14—C10	122.1 (2)
O3—Cu1—O2	88.87 (6)	N4—C14—C6	116.8 (2)
N3—Cu1—O2	91.42 (7)	C10—C14—C6	121.1 (2)
N4—Cu1—O2	90.51 (7)	O8—Cu2—O7	88.94 (8)
N1—Cu1—O2	94.20 (7)	O8—Cu2—O5	89.35 (7)
C15—O2—Cu1	121.65 (14)	O7—Cu2—O5	169.04 (7)
C15—O2—H2O	109.0 (19)	O8—Cu2—O6	168.74 (7)
Cu1—O2—H2O	105.5 (18)	O7—Cu2—O6	91.16 (7)
O2—C15—H15C	109.5	O5—Cu2—O6	88.42 (7)
O2—C15—H15B	109.5	O8—Cu2—O4	102.95 (6)
H15C—C15—H15B	109.5	O7—Cu2—O4	92.05 (7)
O2—C15—H15A	109.5	O5—Cu2—O4	98.88 (7)
H15C—C15—H15A	109.5	O6—Cu2—O4	88.31 (6)
H15B—C15—H15A	109.5	O8—Cu2—Cu2^i^	86.81 (5)
C4—N1—C1	118.3 (2)	O7—Cu2—Cu2^i^	82.29 (5)
C4—N1—Cu1	129.20 (16)	O5—Cu2—Cu2^i^	86.81 (5)
C1—N1—Cu1	112.44 (15)	O6—Cu2—Cu2^i^	82.04 (5)
C3—N2—C2	116.5 (2)	O4—Cu2—Cu2^i^	168.68 (5)
C5—N3—C6	125.59 (19)	C16—O3—Cu1	124.70 (15)
C5—N3—Cu1	118.94 (16)	C16—O4—Cu2	135.91 (14)
C6—N3—Cu1	115.44 (14)	C18—O5—Cu2	119.47 (16)
C13—N4—C14	118.3 (2)	C20—O6—Cu2	125.71 (15)
C13—N4—Cu1	129.49 (17)	C18^i^—O7—Cu2	125.03 (15)
C14—N4—Cu1	112.08 (15)	C20^i^—O8—Cu2	120.48 (15)
N1—C1—C2	120.3 (2)	O4—C16—O3	123.9 (2)
N1—C1—C5	116.22 (19)	O4—C16—C17	120.1 (2)
C2—C1—C5	123.5 (2)	O3—C16—C17	116.0 (2)
N2—C2—C1	122.1 (2)	C16—C17—H17A	109.5
N2—C2—H2	118.9	C16—C17—H17B	109.5
C1—C2—H2	118.9	H17A—C17—H17B	109.5
N2—C3—C4	122.2 (2)	C16—C17—H17C	109.5
N2—C3—H3	118.9	H17A—C17—H17C	109.5
C4—C3—H3	118.9	H17B—C17—H17C	109.5
N1—C4—C3	120.7 (2)	O7^i^—C18—O5	126.2 (2)
N1—C4—H4	119.6	O7^i^—C18—C19	116.8 (2)
C3—C4—H4	119.6	O5—C18—C19	117.0 (2)
O1—C5—N3	128.5 (2)	C18—C19—H19A	109.5
O1—C5—C1	120.2 (2)	C18—C19—H19B	109.5
N3—C5—C1	111.34 (18)	H19A—C19—H19B	109.5
C7—C6—N3	128.8 (2)	C18—C19—H19C	109.5
C7—C6—C14	118.3 (2)	H19A—C19—H19C	109.5
N3—C6—C14	112.89 (19)	H19B—C19—H19C	109.5
C6—C7—C8	120.5 (2)	O6—C20—O8^i^	124.8 (2)
C6—C7—H7	119.8	O6—C20—C21	118.0 (2)
C8—C7—H7	119.8	O8^i^—C20—C21	117.2 (2)
C9—C8—C7	121.8 (2)	C20—C21—H21C	109.5
C9—C8—H8	119.1	C20—C21—H21B	109.5
C7—C8—H8	119.1	H21C—C21—H21B	109.5
C8—C9—C10	119.6 (2)	C20—C21—H21A	109.5
C8—C9—H9	120.2	H21C—C21—H21A	109.5
C10—C9—H9	120.2	H21B—C21—H21A	109.5
C14—C10—C11	117.6 (2)	C22—O9—H9O	109.5
C14—C10—C9	118.7 (2)	O9—C22—H22A	109.5
C11—C10—C9	123.6 (2)	O9—C22—H22B	109.5
C12—C11—C10	119.5 (2)	H22A—C22—H22B	109.5
C12—C11—H11	120.3	O9—C22—H22C	109.5
C10—C11—H11	120.3	H22A—C22—H22C	109.5
C11—C12—C13	119.4 (2)	H22B—C22—H22C	109.5
			
C4—N1—C1—C2	1.0 (3)	C8—C9—C10—C11	−179.5 (2)
Cu1—N1—C1—C2	−175.58 (16)	C14—C10—C11—C12	−1.5 (3)
C4—N1—C1—C5	−179.55 (19)	C9—C10—C11—C12	178.1 (2)
Cu1—N1—C1—C5	3.9 (2)	C10—C11—C12—C13	0.3 (4)
C3—N2—C2—C1	−1.6 (3)	C14—N4—C13—C12	−2.0 (3)
N1—C1—C2—N2	0.5 (3)	Cu1—N4—C13—C12	173.02 (17)
C5—C1—C2—N2	−178.9 (2)	C11—C12—C13—N4	1.5 (4)
C2—N2—C3—C4	1.3 (3)	C13—N4—C14—C10	0.7 (3)
C1—N1—C4—C3	−1.4 (3)	Cu1—N4—C14—C10	−175.18 (16)
Cu1—N1—C4—C3	174.59 (16)	C13—N4—C14—C6	−178.42 (19)
N2—C3—C4—N1	0.2 (4)	Cu1—N4—C14—C6	5.8 (2)
C6—N3—C5—O1	−0.8 (4)	C11—C10—C14—N4	1.0 (3)
Cu1—N3—C5—O1	−178.55 (18)	C9—C10—C14—N4	−178.6 (2)
C6—N3—C5—C1	179.67 (18)	C11—C10—C14—C6	−179.94 (19)
Cu1—N3—C5—C1	1.9 (2)	C9—C10—C14—C6	0.4 (3)
N1—C1—C5—O1	176.57 (19)	C7—C6—C14—N4	178.15 (19)
C2—C1—C5—O1	−4.0 (3)	N3—C6—C14—N4	−0.7 (3)
N1—C1—C5—N3	−3.8 (3)	C7—C6—C14—C10	−0.9 (3)
C2—C1—C5—N3	175.59 (19)	N3—C6—C14—C10	−179.77 (18)
C5—N3—C6—C7	−1.5 (4)	Cu2—O4—C16—O3	176.52 (15)
Cu1—N3—C6—C7	176.32 (18)	Cu2—O4—C16—C17	−2.7 (3)
C5—N3—C6—C14	177.16 (19)	Cu1—O3—C16—O4	−9.5 (3)
Cu1—N3—C6—C14	−5.0 (2)	Cu1—O3—C16—C17	169.72 (17)
N3—C6—C7—C8	179.5 (2)	Cu2—O5—C18—O7^i^	−6.0 (3)
C14—C6—C7—C8	0.8 (3)	Cu2—O5—C18—C19	173.09 (16)
C6—C7—C8—C9	−0.3 (4)	Cu2—O6—C20—O8^i^	2.5 (3)
C7—C8—C9—C10	−0.2 (4)	Cu2—O6—C20—C21	−179.18 (15)
C8—C9—C10—C14	0.1 (3)		

Symmetry code: (i) −*x*+1, −*y*, −*z*+1.

### Hexa-µ-acetato-1:2κ2O:O';2:3κ8O:O'; 3:4κ2O:O'-dimethanol-1κO,2κO-bis[N-(quinolin-8-yl)pyrazine-2-carboxamide]-1κ3N,N',N''; 4κ3N,N',N''-tetracopper(II) methanol disolvate (I) Hydrogen-bond geometry (Å, º)

**Table:**

*D*—H···*A*	*D*—H	H···*A*	*D*···*A*	*D*—H···*A*
O2—H2*O*···O1^ii^	0.86 (3)	1.84 (3)	2.689 (3)	178 (3)
O9—H9*O*···O4	0.84	2.33	2.955 (3)	132
O9—H9*O*···O6	0.84	2.30	3.000 (3)	141
C3—H3···O8^iii^	0.95	2.56	3.508 (3)	174
C7—H7···O1	0.95	2.36	2.943 (3)	119
C9—H9···O9^iv^	0.95	2.57	3.415 (3)	149
C13—H13···O4	0.95	2.54	3.175 (3)	124
C21—H21*C*···O8^v^	0.98	2.59	3.563 (3)	170

Symmetry codes: (ii) −*x*+1, −*y*+1, −*z*; (iii) −*x*+1, −*y*+1, −*z*+1; (iv) −*x*+1, −*y*, −*z*; (v) *x*+1, *y*, *z*.
