# Supplementary material for: The crystal structures of the ligand N-(quinolin-8-yl)pyrazine-2-carboxamide and of a tetra­nuclear copper(II) complex
Source: Acta Crystallogr E Crystallogr Commun. 2019 May 10;75(Pt 6):755–61. doi: 10.1107/S2056989019005450 (PMC6658947; doi:10.1107/S2056989019005450)

Search Overview

Search:	search2
Date/Time done:	Mon Apr 15 09:10:42 2019
Database(s):	CSD version 5.40 updates (Feb 2019)<br>CSD version 5.40 (November 2018)
Restriction Info:	No refcode restrictions applied
Filters:	None
Percentage Completed:	100%
Number of Hits:	15

Single query used. Search found structures that:

match

Query 1

Query 1

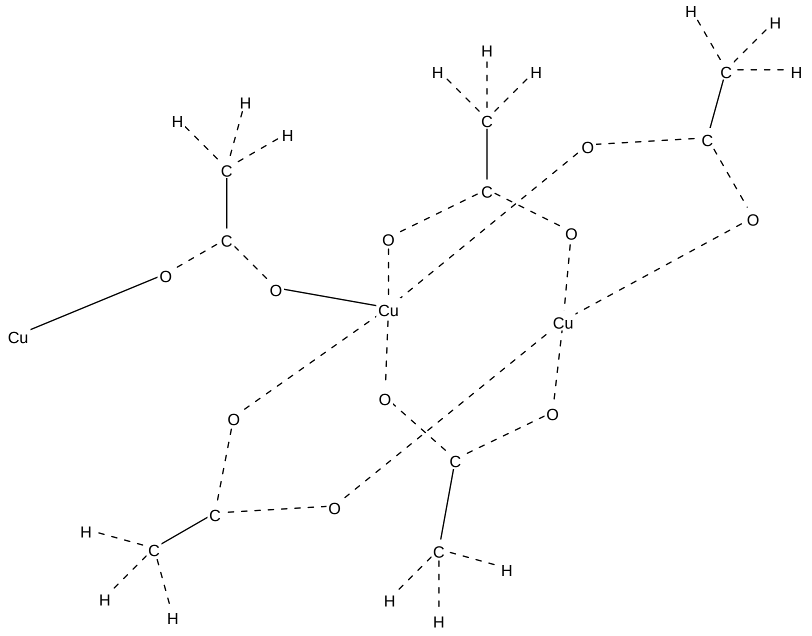

Search: search2 (Mon Apr 15 09:10:42 2019): Hits 1-4

BAHZOZ

Reference: R.L.Beddoes, J.A.Conner, D.Dubowski, A.C.Jones, O.S.Mills, R.Price (1981) *J.Chem.Soc.,Dalton Trans.* ,2119

Formula: $(C_{22}\ H_{45}\ Cu_3\ O_{16}\ P_2)n$

Compound Name: catena-(bis(μ$_3$-Acetato-O,O,O')-tris(μ$_2$-acetato-O,O')-bis(triethylphosphite)-tri-copper(i,ii))

Space Group:	Cc	***Cell:***	***a*** 13.770(40)	***b*** 19.100(30)	***c*** 17.080(40)
Space Group No.:	9	***(Å,°)***	α 90.00	β 125.60(20)	γ 90.00
R-Factor (%):	10.00	***Temperature(K):*** 295	***Density(g/cm^3):*** 1.488		

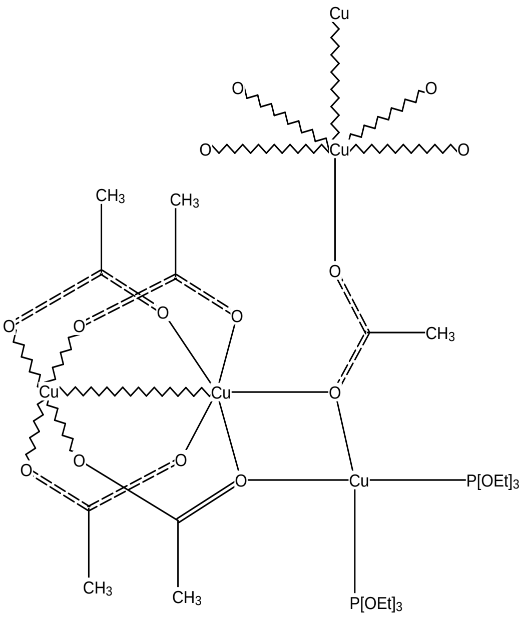

CERTOI

Reference: M.Koman, D.Valigura, E.Durcanska, G.Ondrejovic (1984) *Chem.Commun.* ,381

Formula: $C_{84}\ H_{78}\ Cu_4\ O_{12}\ P_4$

Compound Name: bis(μ$_2$-Acetato-O,O,O')-(tetrakis(μ$_2$-acetato-O,O')-di-copper(ii))-tetrakis(triphenylphosphine)-di-copper(i)

Space Group:	P-1	***Cell:***	***a*** 10.898(8)	***b*** 12.850(4)	***c*** 19.877(8)
Space Group No.:	2	***(Å,°)***	α 121.03(2)	β 90.14(5)	γ 117.98(4)
R-Factor (%):	5.60	***Temperature(K):*** 295	***Density(g/cm^3):*** 1.381		

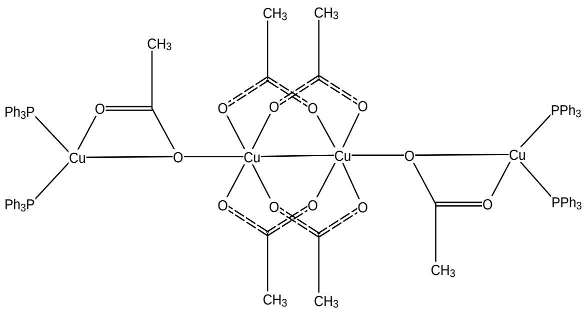

CERTOI10

Reference: D.Valigura, M.Koman, E.Durcanska, G.Ondrejovic, J.Mrozinski (1986) *J.Chem.Soc.,Dalton Trans.* ,2339

Formula: $C_{84}\ H_{78}\ Cu_4\ O_{12}\ P_4$

Compound Name: tetrakis(μ$_2$-Acetato-O,O')-bis(μ$_2$-acetato-O,O,O')-tetrakis(triphenylphosphine-P)-di-copper(i)-di-copper(ii)

Space Group:	P-1	***Cell:***	***a*** 10.898(8)	***b*** 12.850(4)	***c*** 19.877(8)
Space Group No.:	2	***(Å,°)***	α 121.03(2)	β 90.14(5)	γ 117.98(4)
R-Factor (%):	5.65	***Temperature(K):*** 295	***Density(g/cm^3):*** 1.381		

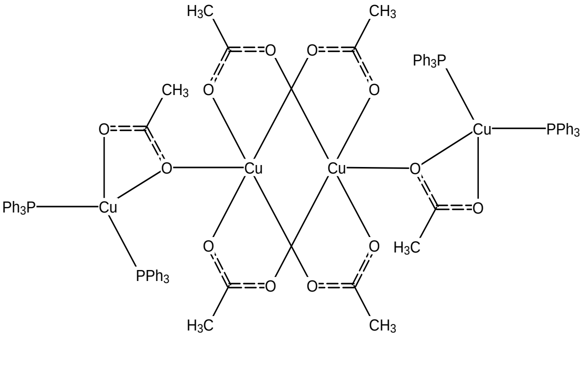

COSQAC

Reference: H.Ackermann, G.Geiseler, K.Harms, R.Leo, W.Massa, F.Weller, K.Dehnicke (1999) *Z.Anorg.Allg.Chem.* ,**625,**1500

Formula: $(C_{32}\ H_{62}\ Cu_5\ N_2\ O_{20}\ P_2)n,4n(C_1\ H_2\ Cl_2)$

Compound Name: catena-(decakis(μ$_2$-Acetato)-bis(μ$_2$-triethylphosphaniminato)-penta-copper(ii) dichloromethane solvate)

Space Group:	P21/c	***Cell:***	***a*** 17.612(1)	***b*** 40.745(2)	***c*** 17.333(1)
Space Group No.:	14	***(Å,°)***	α 90.00	β 91.38(1)	γ 90.00
R-Factor (%):	4.13	***Temperature(K):*** 193	***Density(g/cm^3):*** 1.618		

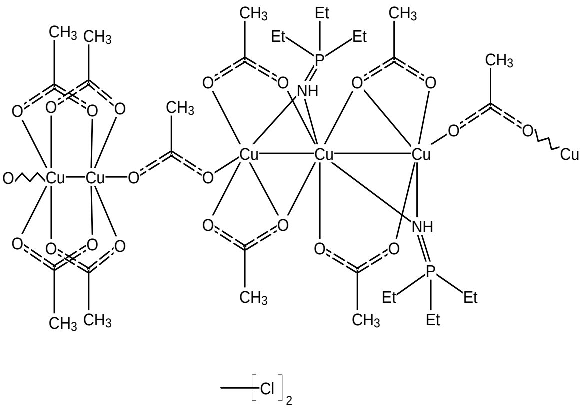

Search: search2 (Mon Apr 15 09:10:42 2019): Hits 5-8

DUHVUZ

Reference: Jingling Zhang, Chunlian Zhang, Yu Xiao, Yan Qin, Shuhua Zhang (2016) *Supramol.Chem.* ,**28,**231

Formula: $(C_{46}\,H_{54}\,Cu_{6}\,N_{10}\,O_{24})n,2(C_{1}\,H_{4}\,O_{1})$

Compound Name: catena-[bis(μ-2,6-di(1H-imidazol-1-yl)pyridine)-dodecakis(μ-acetato)-hexa-copper(ii) methanol solvate]

Space Group:	P-1	***Cell:***	***a***	11.194(0)	***b***	11.292(0)	***c***	12.884(0)
Space Group No.:	2	***(Å,°)***	α	91.33(0)	β	102.48(0)	γ	90.51(0)

R-Factor (%): 4.63 ***Temperature(K):*** 293 ***Density(g/cm^3):*** 1.647

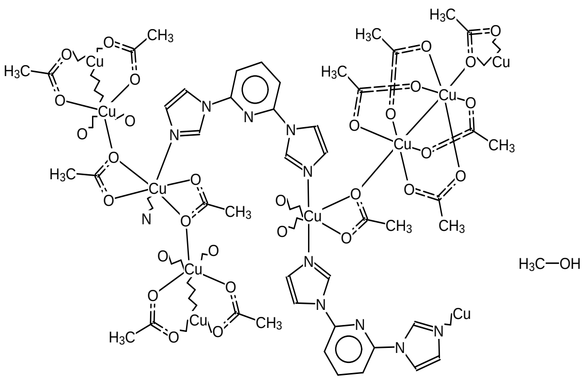

DUQJIK

Reference: M.Fondo, J.Doejo, A.M.Garcia-Deibe, J.Sanmartin, C.Gonzalez-Bello, R.Vicente (2015) *Polyhedron* ,**100,**49

Formula: $C_{28}\,H_{56}\,Cu_{4}\,N_{4}\,O_{20},C_{2}\,H_{3}\,N_{1},2(H_{2}\,O_{1})$

Compound Name: hexakis(μ-acetato)-bis(acetato)-bis(2,2'-(ethane-1,2-diyldiimino) diethanol)-tetra-copper acetonitrile solvate dihydrate

Space Group:	P-1	***Cell:***	***a***	7.930(0)	***b***	11.374(1)	***c***	13.225(1)
Space Group No.:	2	***(Å,°)***	α	95.29(0)	β	98.36(0)	γ	103.02(0)

R-Factor (%): 3.81 ***Temperature(K):*** 100 ***Density(g/cm^3):*** 1.602

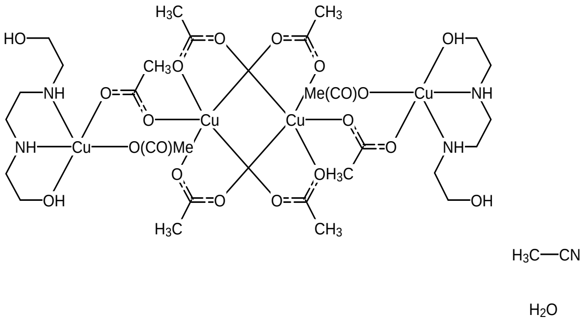

FECQOT

Reference: A.Escuer, S.B.Kumar, M.Font-Bardia, X.Solans, R.Vicente (1999) *Inorg.Chim.Acta* ,**286,**62

Formula: $C_{52}\,H_{48}\,Cu_{4}\,N_{8}\,O_{16}$

Compound Name: bis(μ_2-Acetato-O,O,O')-tetrakis(μ_2-acetato-O,O')-bis(acetato-O)-bis(2,3-bis(2-pyridyl)quinoxaline)-tetra-copper(ii)

Space Group:	C2/m	***Cell:***	***a***	9.904(8)	***b***	13.907(2)	***c***	19.700(3)
Space Group No.:	12	***(Å,°)***	α	90.00	β	102.39(3)	γ	90.00

R-Factor (%): 5.37 ***Temperature(K):*** 295 ***Density(g/cm^3):*** 1.623

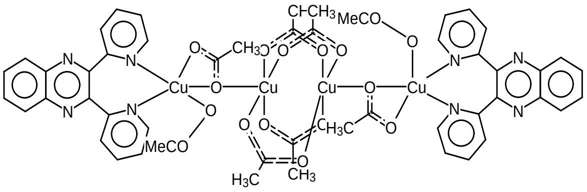

JOZMER

Reference: S.P.Perlepes, E.Libby, W.E.Streib, K.Folting, G.Christou (1992) *Polyhedron* ,**11,**923

Formula: $(C_{36}\,H_{40}\,Cu_{4}\,N_{4}\,O_{16})n$

Compound Name: catena(octakis(μ_2-Acetato)-bis(2,2'-bipyridyl)-tetra-copper(ii))

Space Group:	P-1	***Cell:***	***a***	9.272(2)	***b***	14.718(3)	***c***	8.023(1)
Space Group No.:	2	***(Å,°)***	α	102.66(1)	β	103.89(1)	γ	94.00(1)

R-Factor (%): 3.68 ***Temperature(K):*** 118 ***Density(g/cm^3):*** 1.678

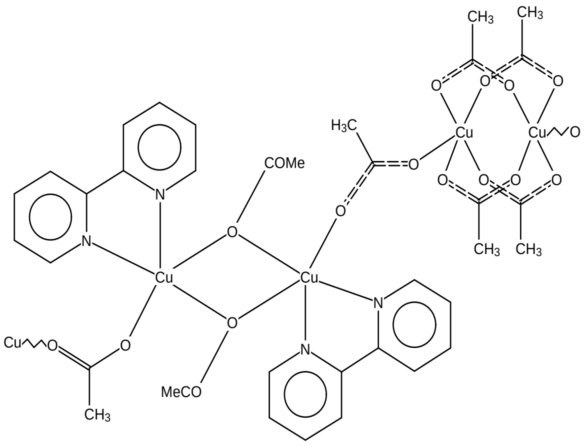

Search: search2 (Mon Apr 15 09:10:42 2019): Hits 9-12

KAQZAE

Reference: Shu-Mei Chen, Can-Zhong Lu, Chang-Kun Xia, Xin-Jiang Xu, Quan-Guo Zhai (2005) *Cryst.Growth Des.* ,**5,**1485

Formula: $(C_{36} H_{34} Cu_3 N_2 O_{14})n$

Compound Name: catena-(hexakis(μ_2-acetato)-(1,4-bis(2-(5-phenyloxazolyl))benzene)-tri-copper(i,ii))

Space Group:	P-1	***Cell:***	***a*** 9.575(0)	***b*** 10.485(0)	***c*** 10.830(0)
Space Group No.:	2	***(Å,°)***	α 113.69(0)	β 94.92(0)	γ 110.59(0)

R-Factor (%): 8.08 ***Temperature(K):*** 293 ***Density(g/cm^3):*** 1.680

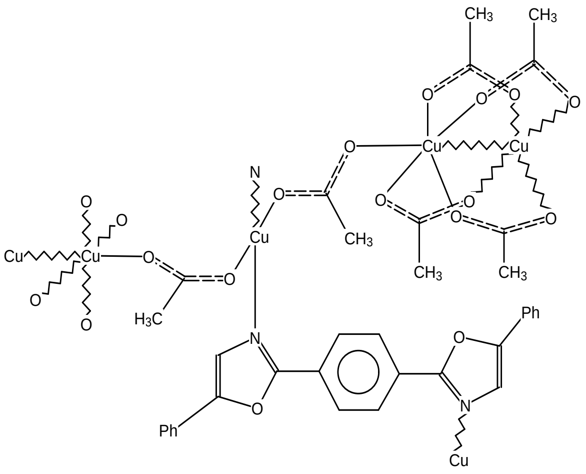

PIBXOU

Reference: B.Chiari, O.Piovesana, T.Tarantelli, P.F.Zanazzi (1993) *Inorg.Chem.* ,**32,**4834

Formula: $C_{44} H_{62} Br_2 Cu_4 N_4 O_{14}$

Compound Name: hexakis(μ_2-Acetato)-bis(1-(5-bromosalicylaldimino)-3-(2-methylpiperidino)propane)-tetra-copper

Space Group:	P21/n	***Cell:***	***a*** 11.627(3)	***b*** 22.442(3)	***c*** 9.693(3)
Space Group No.:	14	***(Å,°)***	α 90.00	β 94.46(2)	γ 90.00

R-Factor (%): 7.50 ***Temperature(K):*** 295 ***Density(g/cm^3):*** 1.692

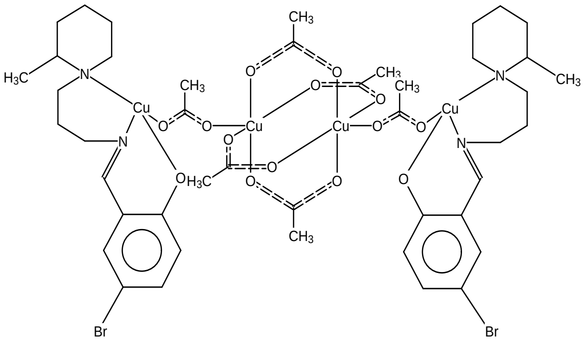

PIMBIW

Reference: J.Valo, M.Nasakkala (1994) *Acta Chem.Scand.* ,**48,**20

Formula: $(K_1{}^{1+})2n,n(C_{16} H_{24} Cu_3 O_{16}{}^{2-})$

Compound Name: catena-(Di-potassium hexakis(μ_2-acetato-O,O')-bis(acetato-O)-tri-copper(ii))

Space Group:	C2/c	***Cell:***	***a*** 17.956(4)	***b*** 14.313(3)	***c*** 12.443(2)
Space Group No.:	15	***(Å,°)***	α 90.00	β 98.85(3)	γ 90.00

R-Factor (%): 3.80 ***Temperature(K):*** 295 ***Density(g/cm^3):*** 1.558

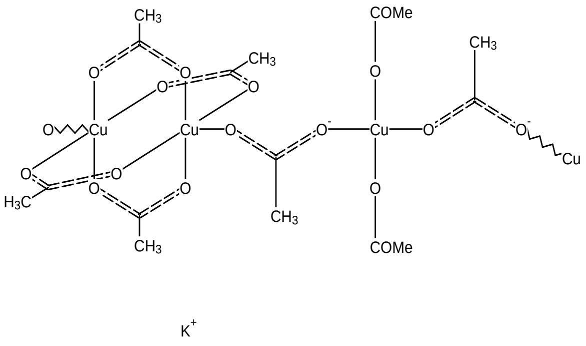

SUJXUS

Reference: P.Niedziejko, M.Szewczyk, P.Kalicki, Z.Kaluza (2015) *Tetrahedron:Asymm.* ,**26,**1083

Formula: $C_{46} H_{68} Cu_4 N_4 O_{16},2(C_1 H_2 Cl_2)$

Compound Name: hexakis(μ-acetato)-diacetato-bis(1-(phenyl(pyrrolidin-2-yl)methyl) pyrrolidine)-tetra-copper dichloromethane solvate

Space Group:	P21212	***Cell:***	***a*** 14.126(0)	***b*** 24.020(0)	***c*** 9.814(0)
Space Group No.:	18	***(Å,°)***	α 90.00	β 90.00	γ 90.00

R-Factor (%): 4.20 ***Temperature(K):*** 296 ***Density(g/cm^3):*** 1.354

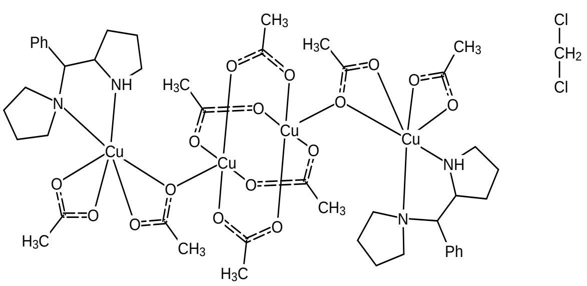

Search: search2 (Mon Apr 15 09:10:42 2019): Hits 13-15

UJOWEX

Reference: G.Huang, X.Liu (2016)
Acta Crystallogr.,Sect.E:Cryst.Commun. ,**72,**597

Formula: $C_{44}\ H_{64}\ Cu_4\ N_4\ O_{14}$

Compound Name: hexakis(μ_2-Acetato)-bis(2-(((2,2,6,6-tetramethylpiperidin-4-yl)imino) methyl)phenolato)-tetra-copper(ii)

Space Group:	Pbcn	***Cell:***	***a***	31.243(0)	***b***	10.787(0)	***c***	15.256(0)
Space Group No.:	60	***(Å,°)***	α	90.00	β	90.00	γ	90.00

R-Factor (%): 3.80 ***Temperature(K):*** 250 ***Density(g/cm^3):*** 1.456

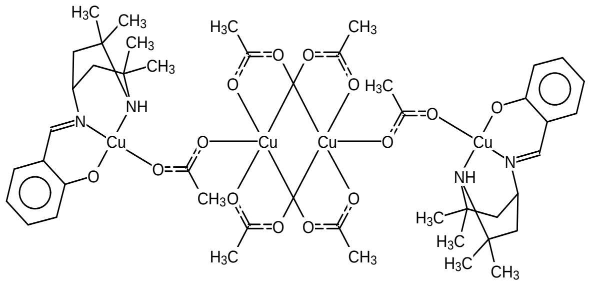

YEKTAJ

Reference: C.A.Crawford, E.F.Day, W.E.Streib, J.C.Huffman, G.Christou (1994) *Polyhedron* ,**13,**2933

Formula: $(C_{28}\ H_{40}\ Cu_4\ N_4\ O_{16})n$

Compound Name: catena-(hexakis(μ_2-Acetato-O,O')-bis(μ_2-acetato-O,O)-bis(2-aminomethylpyridine-N,N')-tetra-copper(ii))

Space Group:	P-1	***Cell:***	***a***	8.522(1)	***b***	13.066(2)	***c***	7.942(1)
Space Group No.:	2	***(Å,°)***	α	99.22(1)	β	90.73(1)	γ	97.66(1)

R-Factor (%): 2.75 ***Temperature(K):*** 104 ***Density(g/cm^3):*** 1.811

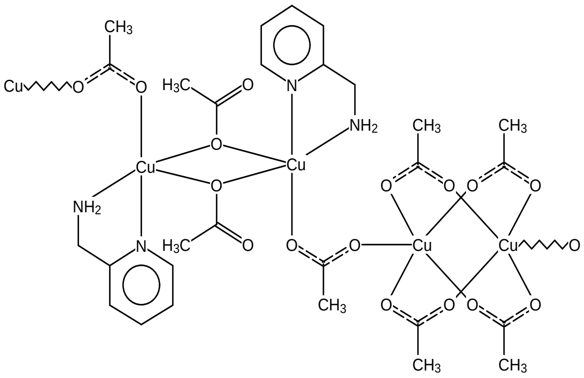

YOMTUP

Reference: A.Neels, H.Stoeckli-Evans, A.Escuer, R.Vi (1995)
Inorg.Chem. ,**34,**1946

Formula: $(C_{30}\ H_{34}\ Cu_4\ N_4\ O_{16})n$

Compound Name: catena-(octakis(μ_2-Acetato-O,O')-(μ_2-2,5-bis(2-pyridyl)pyrazine-N,N',N'',N''')-tetra-copper(ii))

Space Group:	P-1	***Cell:***	***a***	7.916(1)	***b***	9.280(3)	***c***	12.732(4)
Space Group No.:	2	***(Å,°)***	α	95.49(1)	β	90.06(1)	γ	106.57(1)

R-Factor (%): 4.40 ***Temperature(K):*** 295 ***Density(g/cm^3):*** 1.789

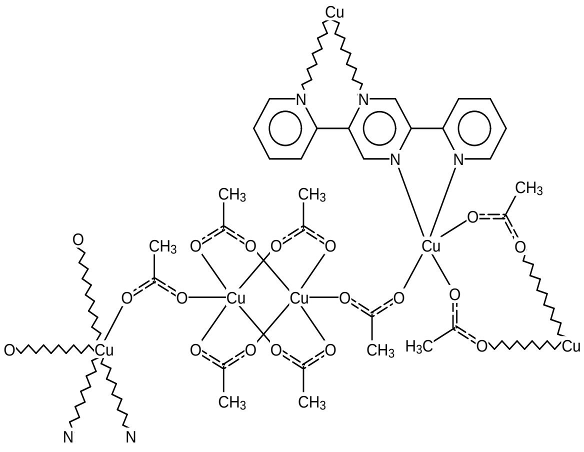

Supplement: Supplementary file 4 [file e-75-00755-sup4.pdf]
